# PD–DEM hybrid modeling of leading edge erosion in wind turbine blades under controlled impact scenarios

**DOI:** 10.1007/s40571-024-00717-y

**Published:** 2024-02-22

**Authors:** Khuram Walayat, Sina Haeri, Imran Iqbal, Yonghao Zhang

**Affiliations:** 1 Institute for Materials and Processes, School of Engineering, University of Edinburgh, Sanderson Building, King’s Buildings, Robert Stevenson Road, Edinburgh, EH9 3FB UK; 2 HR Wallingford, Howbery Park, Wallingford, Oxfordshire UK; 3 Department of Information and Computational Sciences, School of Mathematical Sciences and LMAM, Peking University, Beijing, 100871 China; 4 Institute of Mechanics, Chinese Academy of Sciences, Beijing, 100190 China

**Keywords:** Peridynamics, Discrete element method, Wind turbine blades, Leading edge erosion, Solid particle erosion, Damage, Crack

## Abstract

This paper addresses the critical issue of leading edge erosion (LEE) on modern wind turbine blades (WTBs) caused by solid particle impacts. LEE can harm the structural integrity and aerodynamic performance of WTBs, leading to reduced efficiency and increased maintenance costs. This study employs a novel particle-based approach called hybrid peridynamics–discrete element method (PD–DEM) to model the impact of solid particles on WTB leading edges and target material failure accurately. It effectively captures the through-thickness force absorption and the propagation of stresses within the leading edge coating system composed of composite laminates. The amount of mass removed and the mean displacement of the target material points can be reliably calculated using the current method. Through a series of tests, the research demonstrates the method’s ability to predict impact force changes with varying particle size, velocity, impact angles and positions. Moreover, this study offers a significant improvement in erosion prediction capability and the development of design specifications. This work contributes to the advancement of WTB design and maintenance practices to mitigate LEE effectively.

## Introduction

The global demand for renewable energy has accelerated the exploitation of sustainable sources including wind, hydro, wave and solar to generate electricity. Wind energy, harnessed by wind turbines, is one of the renewable resources which is relatively reliable if the installation site is carefully chosen [1, 2], resulting in accelerated adoption of the technology over the past two decades [3]. Significant challenges exist in the further development of wind energy due to uncertain and often harsh environmental conditions encountered in installation sites. The efficiency of a wind turbine is mainly dependent on the aerodynamic performance of the blades which is gradually degraded by leading edge erosion (LEE). LEE is typically accelerated by exposure to harsh environmental conditions such as temperature fluctuations, moisture and UV radiation. However, it is primarily initiated due to the accumulation of micro-damages resulting from the impact of raindrops, sand, hailstones or other particles on the leading edge of the blade.

The modern turbine blades move at a speed of well above 80 m/s as they are naturally exposed to high-speed winds and are often subjected to the impacts of raindrops and solid particles (e.g., hailstones or sand) [4]. The particles may cause abrasive wear or impact erosion depending on their size and angle of impact. When a particle hits the blade surface, the contact pressure causes waves to propagate through the protective layers which leads to the initiation of damage, deterioration of the materials, fatigue, coating cracking, debonding, cracks in the composite and surface roughening [5].

Initially, the increasing surface roughness raises the friction drag, leading to an earlier stall onset, which significantly reduces aerodynamic efficiency. Ultimately, as the coating of the blade is compromised, synergy with UV degradation and other corrosives speeds up the erosion rate leading to widespread damage to internal structures, resulting in unexpected downtime and significant maintenance costs [6]. Therefore, it is crucial to consider the erosive impact of particles as a part of the design cycle of the blades. The complex nature of LEE involves the interplay of aerodynamics, mechanics of multilayered materials and composites, impact dynamics and fatigue [6], which underscores the need for the development of new simulation techniques to better understand and model this phenomenon. In operation, wind turbine blades can be monitored for degradation through visual inspection or video monitoring from a fixed point or with drones [7], but these approaches have limitations and offer a low resolution. In laboratory experiments, nondestructive methods such as X-ray tomography can be used to scan test specimens and observe the damage caused by impacting particles [7]. For design, rain erosion rigs [8] and the single-point impact fatigue test [9] are the common methods to investigate erosion in wind turbine blades which are expensive and need specialized designs.

There are plenty of empirical erosion models [10–13] in the literature, which are often specific to particular materials or conditions. Empirical models rely on observed data and are simple but limited in applicability. The development of numerical models is another approach for investigating the LEE process which can lead to affordable and rapid design tools. In contrast to empirical models, numerical erosion models employ complex mathematical simulations to capture erosion phenomena comprehensively, making them versatile but computationally demanding. Numerous attempts have been made in the past to model the LEE [14–19]. Most of the research has been carried out for modeling LEE brought on by raindrops [20]. In the most recent attempts to model droplet impact erosion on leading edges [14–16], the fatigue approach has been employed by establishing a stress state to account for damage accumulation.

There has been limited research on modeling the erosion caused by solid particle impact on WTB [21, 22]. Although the fundamental principle of impact erosion is the same across several industrial applications, the circumstances and materials differ significantly. Different numerical methods have been employed to model the solid particle erosion (SPE) [23–32] of various materials due to single and multiple impacts, such as the finite element method (FEM) [23, 24, 30, 33], smoothed particle hydrodynamics (SPH) [25, 26] and finite volume particle method (FVPM) [32]. These models have been used to investigate the effect of various parameters, including impact angles, speeds and particle shape, on the erosion mechanisms and mass removal rates. Additionally, computational fluid dynamics and discrete phase model (CFD-DPM) [34] have been applied to simulate the erosion caused by sand particles on the blades of tidal current turbines.

Among these methods, FEM is quite effective in predicting stress and strain fields; however, it faces difficulties in modeling crack initiation and propagation. Meshless techniques such as SPH also face limitations for modeling fracture initiation and propagation as it is fundamentally assumed the body remains a continuum as it deforms. To address these issues, peridynamics (PD) was introduced as a non-local continuum mechanics theory by Silling [35]. Material points within a defined horizon radius affect each other’s state, and damage is considered a material response in PD theory [36]. Damage can initiate at multiple points and spread through internal structures, without using any specific crack development criterion. The PD theory has been effectively applied to material deformation and impact damage prediction applications [37–39]. In PD, rigid impactors or short-range repulsive force algorithms are usually employed to simulate the impact. However, in most of the investigations, the penalty stiffness or short-range force constants are arbitrarily chosen, or the formulations are unrealistic. Furthermore, the tangential forces, friction and restitution coefficient cannot be directly incorporated into the formulation. Therefore, the current PD models may not be able to accurately evaluate the impact forces on the surface which is extremely important to predict the erosive impact of particles on the material.

A generic contact modeling strategy for impact problems by coupling PD with the discrete element method (DEM) has recently been introduced as an alternative to the aforementioned contact models [40–47]. The DEM [48] has been proved as a standard technique for simulating collision processes between distinct solid bodies. Although DEM can describe the interaction between solid objects [48], it cannot model particle deformation, particularly particle damage. The ad hoc extensions to DEM so far lack a general mathematical framework to consider different materials (e.g., ductile and brittle behavior), whereas a coupled PD–DEM scheme combines the distinct strengths of PD and DEM, which allows for the generation and adjustment of appropriate contact forces in both the normal and tangential directions while capturing the damage.

The objective of this study is to use our hybrid PD–DEM method [49], which has recently been developed within the particle-based LAMMPS^1^ framework, for modeling SPE of the leading edge of WTB. We used bond-based prototype micro-elastic brittle (PMB) material model with constant horizon to predict deformation and impact damage in brittle material. However, there are several bond-based and state-based peridynamics models that can be adapted for simulating ductile materials. For ductile materials, micro-plastic (MP) model can be used to simulate impact damage. The DEM contact models can provide the appropriate contact forces, damping effects and intra-particle stiffness by adjusting the contact parameters. In the current investigations, the tangential contact force is calculated using the Mindlin’s stick–slip friction model [50], and the normal contact force is estimated using the Hertzian force–displacement law [51]. By considering the contact mechanics directly, the hybrid PD–DEM avoids arbitrary selection of the penalty parameters which is a common practice in PD contact models for impact event simulations. Additionally, these contact laws are extendable for systems involving simultaneous multi-particle interactions [52, 53]. In our previous work [49], the hybrid PD–DEM has already been rigorously validated for the contact parameters, predicted damage patterns and material loss. Here, we used the model to study the erosion caused by impinging sand particles at the leading edge of a WTB and analyze the effects of erosive particle-related factors such as particle size, impact velocity, impact angle and impact position.

## Mathematical model

The mathematical framework for hybrid PD–DEM has been established in our earlier work [49]. In this section, we present a concise overview of our model.

### Peridynamics formulation

**Fig. 1:**
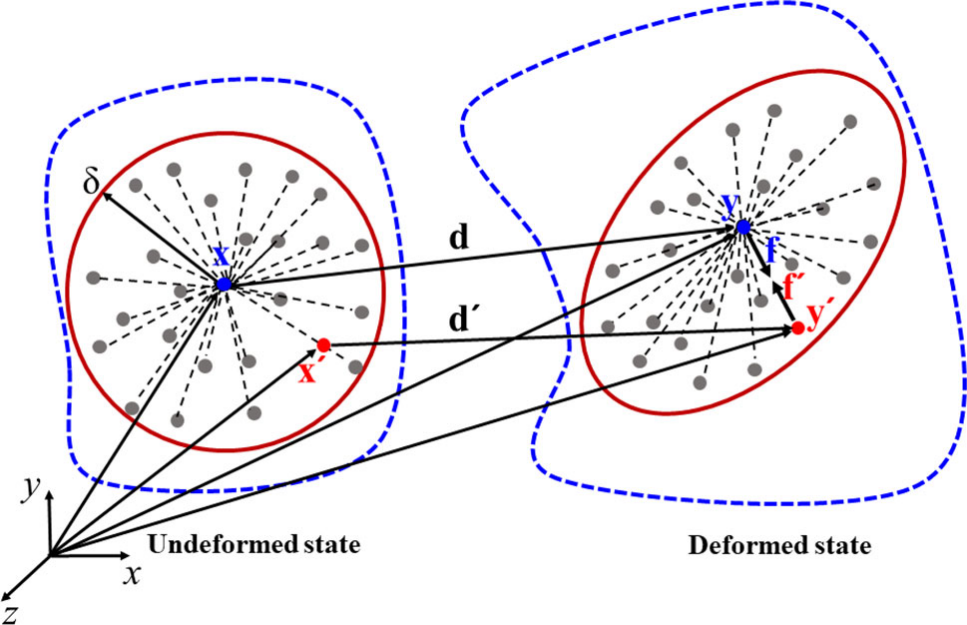
Bond-based PD involves the interaction between material points ${\mathbf{x}}$ and ${\mathbf{x^{\prime}}}$, and their corresponding counterparts, material points ${\mathbf{y}}$ and ${\mathbf{y^{\prime}}}$ in undeformed and deformed states, respectively

The peridynamics theory employs integral equations instead of partial differential equations used in the classical continuum mechanics to explain the mutual displacements and non-local exchange of fundamental information through forces applied between material points over finite distances [35]. This approach allows for the spontaneous formation of discontinuities and cracks in continuous materials. The focus of this study is on using the hybrid PD–DEM [49] to simulate leading edge erosion, and a brief description of the bond-based PD theory for brittle material is presented here and the interested reader is referred to [35, 36, 54] for more details. Within the interaction domain $H_{{\mathbf{x}}}$ as depicted in Fig. 1, a material point ${\mathbf{x}}$ in the bond-based PD [35] interacts with another material point ${\mathbf{x^{\prime}}}$. The interaction domain $H_{{\mathbf{x}}}$ of the material point ${\mathbf{x}}$ is assumed to be a spherical region specified by a radius $\delta$ which is known as its horizon. Material points within the interaction domain $H_{{\mathbf{x}}}$ of the material points ${\mathbf{x}}$ are called the family members of ${\mathbf{x}}$.

The PD equation of motion, proposed by Silling et al. [35], governs the interactions between a material point ${\mathbf{x}}$ and another material point ${\mathbf{x^{\prime}}}$ within the interaction domain $H_{{\mathbf{x}}}$, as

1 $$ \rho_{m} {{\ddot{\mathbf{d}}}}({\mathbf{x}},\,\tau ) = \int\limits_{{H_{{\mathbf{x}}} }} {{\mathbf{f}}(\eta ,\,\xi ){\text{d}}V_{{x^{\prime}}} + {\mathbf{F}}_{b} ({\mathbf{x}},\,\tau )} , $$

where the material points are represented by spherical PD particles having a diameter of $d_{m}$. In Eq. (1), $\rho_{m}$ is the density of the PD particle, while **d** represents the displacement vector of a particle situated at the position **x** at a time $\tau$. The derivative of the displacement vector ${{\ddot{\bf d}}}$ for each particle with respect to time is related to the integral of an internal force field **f**(*η*, *ξ*) and an external body force **F**_*b*_ The force applied on a PD particle located at the point **x** by all the PD particles within *H*_x_ is expressed as the integral of a force density **f**(*η*, *ξ*) over the volume *V*_x′_, where *ξ* = **x**′ − **x** and *η* = **d**′ − **d** are the relative position and displacement vectors, respectively. The force density **f**(*η*, *ξ*), which represents inter-particle bonds, is described as

2 $$ {\mathbf{f}}(\eta ,\,\xi ) = \mu cs{\mathbf{n.}} $$

The bond constant $c$, also referred to as the micro-modulus function, is a PD parameter that is determined by equating strain energy densities from the classical theory of elasticity with peridynamics under simple loading conditions [43, 44]. The expression for $c$ is given as

3 $$ c = \frac{15E}{{\pi \delta^{4} (1 + \nu )}}. $$

Here, $E$ is Young’s modulus, while $\nu$ is the Poisson’s ratio of the material. In Eq. (2), **n** is a unit vector that points from **x** + **d** to ** x**′ + **d**′, and the bond stretch is denoted by $s$ is expressed as

4 $$ s = \frac{{\left| {\eta + \xi } \right| - \left| \xi \right|}}{\left| \xi \right|}. $$

**Fig. 2:**
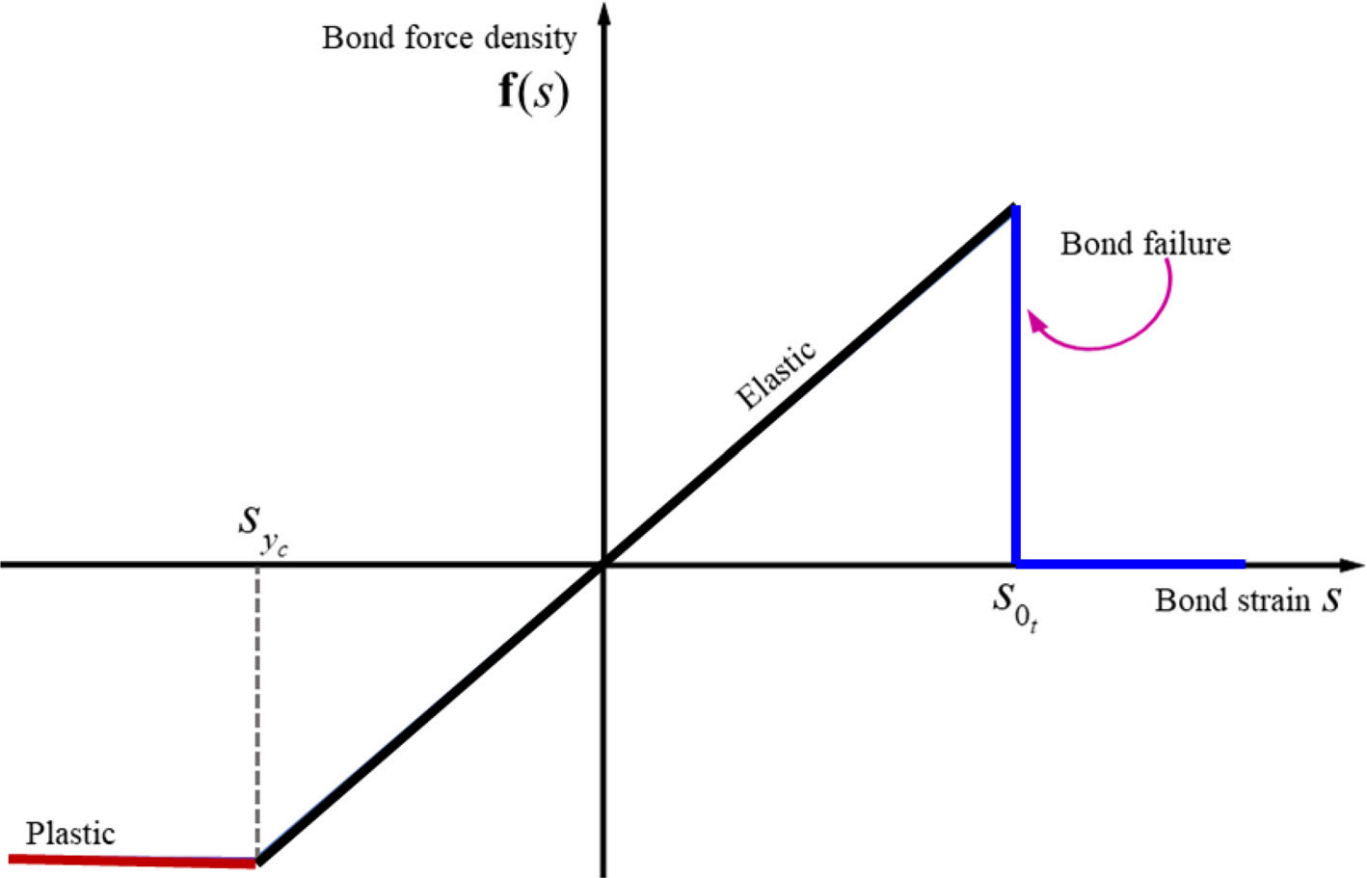
Constitutive bond model

When the value of the bond stretch $s$ exceeds its critical value $s_{{\text{c}}}$, the bond breaks and this is an irreversible process. The critical stretch $s_{{\text{c}}}$ for bond-based PD in 3D was determined by Silling et al. [55] and is expressed as

5 $$ s_{{\text{c}}} = \sqrt {\frac{{10G_{c} }}{{\pi c\delta^{5} }}} , $$

where $G_{c}$ denotes the fracture energy per unit area of the material. In Eq. (2), the parameter $\mu$ is a function dependent on the material’s history of damage and breaks bonds when the stretch $s$ exceeds the critical stretch $s_{0}$. Its value is either 0 or 1; if $s \le 0$, $\mu = 1$; otherwise, $\mu = 0$. Figure 2 shows the elastic and perfectly plastic constitutive model for the bonds. The force density function can be nonzero for both compressive and tensile states. For elastic and plastic regions, the force density relationship can be written as

6 $$ {\mathbf{f}}\left( s \right) = \left\{ {\begin{array}{*{20}l} {cs,} \hfill & {{\text{if}}\,\,s_{{y_{c} }} < s(t) < s_{{0_{t} }} } \hfill \\ {cs_{{y_{c} }} } \hfill & {{\text{if}}\,\,s(t) < s_{{y_{c} }} } \hfill \\ \end{array} } \right.. $$

Silling et al. [55] proposed a method to quantify the local damage at a material point, which ranges from 0 to 1. This can be expressed as a weighted ratio of the number of broken bonds to the total number of initial bonds between the material point and its family members.

7 $$ \phi ({\mathbf{x}},\,\tau ) = 1 - \frac{{\int\limits_{{H_{x} }} {\mu \left( {{\mathbf{x}}\prime - {\mathbf{x}},\,\tau } \right){\text{d}}V^{\prime}} }}{{\int\limits_{{H_{x} }} {{\text{d}}V^{\prime}} }}. $$

When $\phi = 1$, it denotes a completely damaged point, all the bonds initially associated with the material point have been eliminated, and $\phi = 0$ indicates an undamaged material point, i.e., all interactions are intact.

### Solid DEM particle 

The DEM model uses the Newton–Euler equations of rigid body dynamics to govern the translational and rotational motion of solid particles, which are used to simulate the motion of impacting sand particles. The total forces and torques acting on the *j*th particle are summed up in the vectors ${\mathbf{F}}_{j}$ and ${\mathbf{T}}_{j}$, respectively, as

8 $$ {\mathbf{F}}_{j} = \sum\limits_{c = 1}^{{n_{c} }} {{\mathbf{F}}_{j}^{{{\text{ext}}}} + {\mathbf{F}}_{j}^{c} + {\mathbf{F}}_{j}^{{{\text{damp}}}} } , $$

9 $$ {\mathbf{T}}_{j} = \sum\limits_{c = 1}^{{n_{c} }} {\left( {{\mathbf{r}}_{j}^{c} \times {\mathbf{F}}_{j}^{c} + {\mathbf{q}}_{j}^{c} } \right)} + {\mathbf{T}}_{j}^{{{\text{ext}}}} + {\mathbf{T}}_{j}^{{{\text{damp}}}} , $$

where ${\mathbf{F}}_{j}^{c}$ is the contact force due to interaction of the particle $j$ with other particles and obstacles, ${\mathbf{F}}_{j}^{{{\text{ext}}}}$ and ${\mathbf{T}}_{j}^{{{\text{ext}}}}$ are the external load, ${\mathbf{F}}_{j}^{{{\text{damp}}}}$ and ${\mathbf{T}}_{j}^{{{\text{damp}}}}$ are the force and torque because of damping in the system, ${\mathbf{q}}_{j}^{c}$ is the torque other than due to a tangential force, e.g., rolling motion or torsion, ${\mathbf{r}}_{j}^{c}$ is a vector connecting particle center with the contact point, and $n_{c}$ is the total number of particles in contact with the particle $j$.

### Contact model

The primary goal of using coupled PD–DEM is to take advantage of the DEM contact laws. Here, we present a summary of PD coupling with DEM and their interaction; for a comprehensive discussion as well as formulation and evolution of multi-particle contact forces, readers are referred to [49]. The contact approach used in this study is similar to the extensively used DEM method, which employs Hertz’s theory [51] for force–displacement relationships in the normal direction, and no-slip elastic solutions for force–displacement relations proposed by Mindlin [50] in the tangential direction. The normal contact force ${\mathbf{F}}_{n}$ and tangential contact force ${\mathbf{F}}_{t}$ on the particle $i$ due to its interaction with neighboring particles $N_{k}$ become

10 $$ {\mathbf{F}}_{n} = \frac{1}{{N_{k} }}\sum\limits_{j = 1}^{{N_{k} }} {\left( {K_{n} {\mathbf{D}}_{{\text{n}}} + C_{{\text{n}}} {\dot{\mathbf{D}}}_{{\text{n}}} } \right)} ,\,\,\,j = 1,2,3,....,N_{k} $$

11 $$ {\mathbf{F}}_{t} = \frac{1}{{N_{k} }}\sum\limits_{j = 1}^{{N_{k} }} {\left( {K_{t} {\mathbf{D}}_{{\text{t}}} + C_{{\text{t}}} {\dot{\mathbf{D}}}_{{\text{t}}} } \right)} ,\,\,\,j = 1,2,3,....,N_{k} $$

where ${\mathbf{D}}_{{\text{n}}}$ is normal and ${\mathbf{D}}_{{\text{t}}}$ is tangential overlap displacements. $K_{n}$ and $K_{t}$ are stiffness constants, while $C_{{\text{n}}}$ and $C_{{\text{t}}}$ are damping constants in the normal and tangential direction, respectively. To determine the values of these displacements and constants, we use the formulas provided in [49]. A Coulomb friction coefficient $\lambda$ is used to model a stick and slip behavior [56], and ${\mathbf{F}}_{t}$ of two interacting particles $i$ and $j$ is set as

12 $$ {\mathbf{F}}_{t,ij} = \lambda {\mathbf{F}}_{n,ij} $$

The external body force as in Eq. (1) acting on PD particle $i$ becomes

13 $$ {\mathbf{F}}_{b} ({\mathbf{x}}_{i} ,\,\tau ) = {\mathbf{F}}_{(i),n} + {\mathbf{F}}_{(i),t} , $$

while the contact force on the impactor ${\mathbf{F}}_{j}^{c}$ in Eqs. (8) and (9) becomes

14 $$ {\mathbf{F}}_{j}^{c} = - \left( {\frac{{{\mathbf{F}}_{(i),n} + {\mathbf{F}}_{(i),t} }}{{V_{i} N_{i} }}} \right) $$

## Validation tests

This PD–DEM hybrid approach offers a comprehensive and effective solution for simulating particle erosion. Although the accuracy and reliability of the current approach have been demonstrated in our previous work [49], we conduct two validation tests with parameters and problem setup more relevant to the cases considered in this paper.

### Contact force

**Fig. 3:**
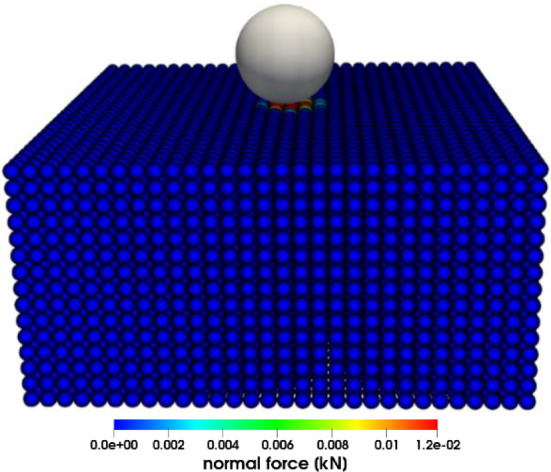
Impact force in the normal direction during the impact event

**Fig. 4:**
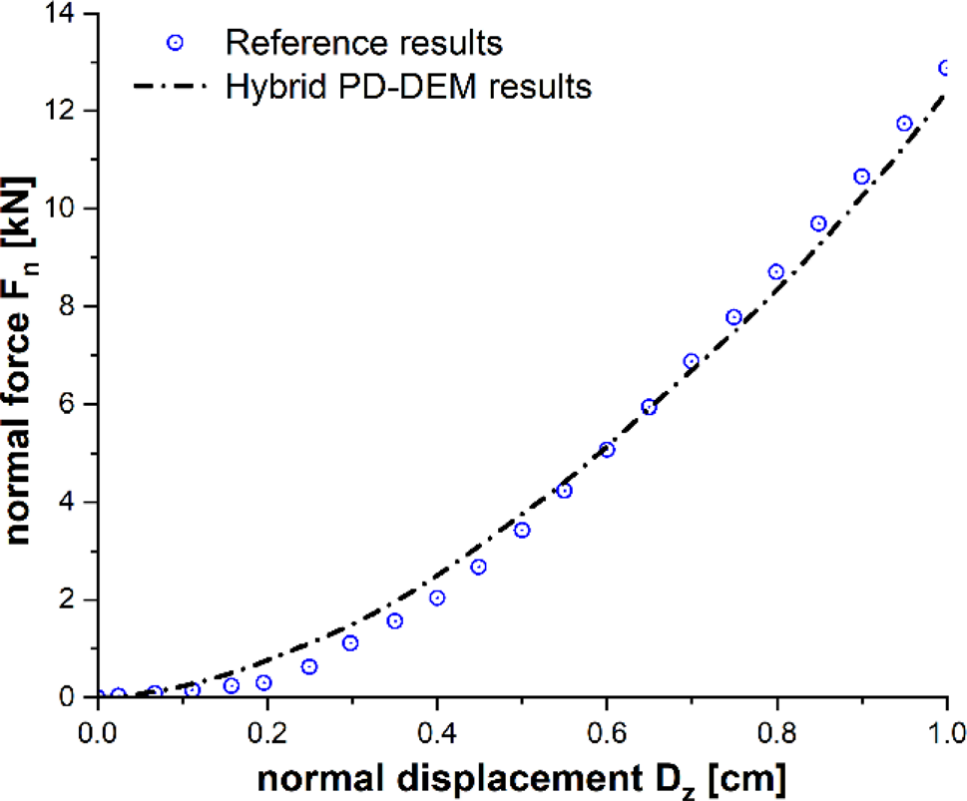
Normal reaction force with respect to the penetration depth of a solid sphere during normal impact on half-space

Here, the solution for the contact between a rigid sphere and an elastic half-space, which was derived from Hertzian contact theory [57, 58], is being examined. A spherical particle of radius $R = 0.05\,m$ described by DEM is projected normally at the center of a half-space with velocity $V_{z} = - 0.01\,\,\,{\text{ms}}^{ - 1}$. The half-space is geometrically defined by length $l = 0.45\,{\text{m}}$, width $w = 0.45\,{\text{m}}$ and thickness $h = 0.225\,{\text{m}}$. The target half-space is discretized with PD particles of radius $r = {0}{\text{.015}}\,{\text{m}}$ as shown in Fig. 3. The interactions between material points of the target plate are represented by the bond-based PD particles. The material parameters of the sphere and target half-space are listed in Table 1. The results obtained using the present hybrid model are plotted in Fig. 4, and the comparison of the obtained data with the results from the literature [58] shows good agreement. Both methods use a similar approach for calculating interaction forces, but the key distinction lies in how they define the interaction between the PD body and DEM particles. In the reference [58], researchers considered imaginary DEM particles on the surface of the PD bodies, while our approach introduces a hybrid PD–DEM potential that uniformly extends throughout the PD bodies. Figure 4 shows the impact force on the half-space in the normal direction after the impacting rigid particle rebounds. We have observed that our results are sensitive to factors such as particle size differences and time step sizes. As the size difference between DEM and PD particles increases, it necessitates the use of smaller time step sizes to accurately compute contact forces. Therefore, we used adaptive time settings to ensure robust and reliable simulations that underscore sensitivity.

**Table 1:** Material properties

Object	Young’s modulus *E* (Pa)	Poisson’s ratio *v*	Density (kg/m^3^)
Sphere	1e8	0.25	1100
Half-space	1e8	0.25	1100

### Erosion rate

**Fig. 5:**
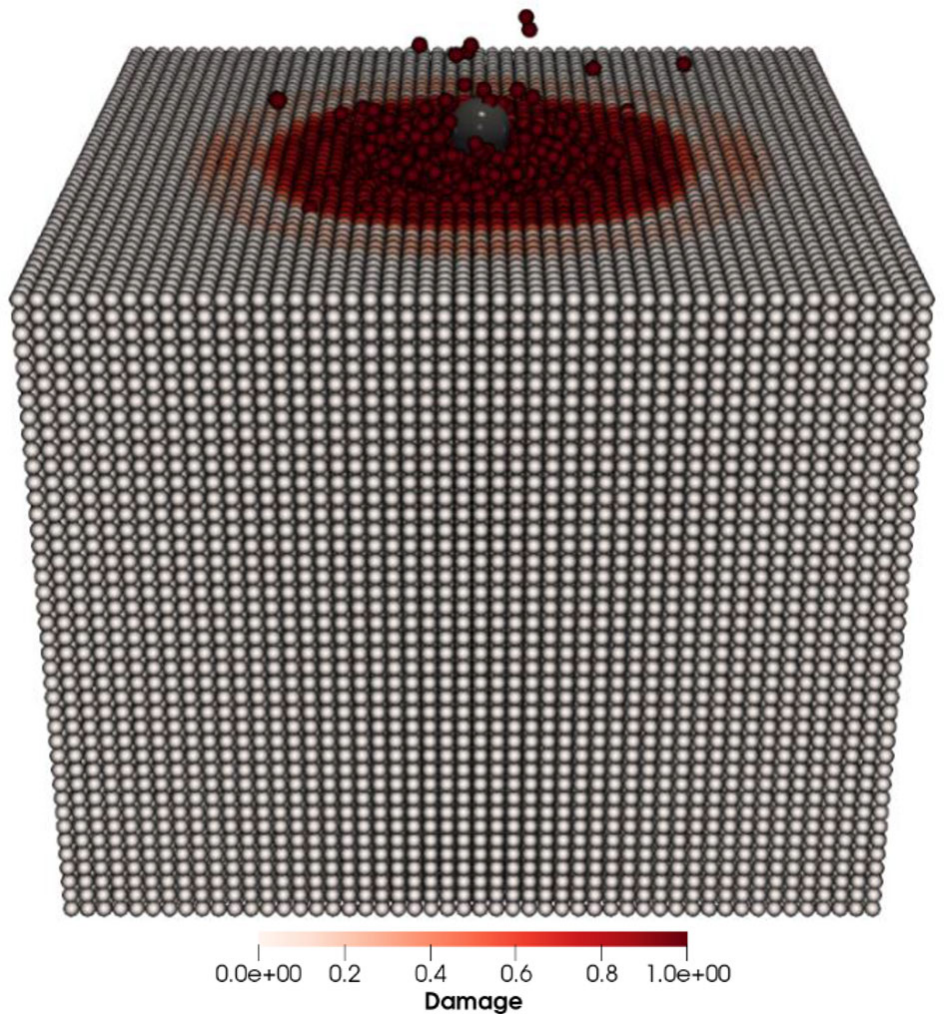
Erosion of target at 45° impingement angle and 55 m/s impact velocity

**Fig. 6:**
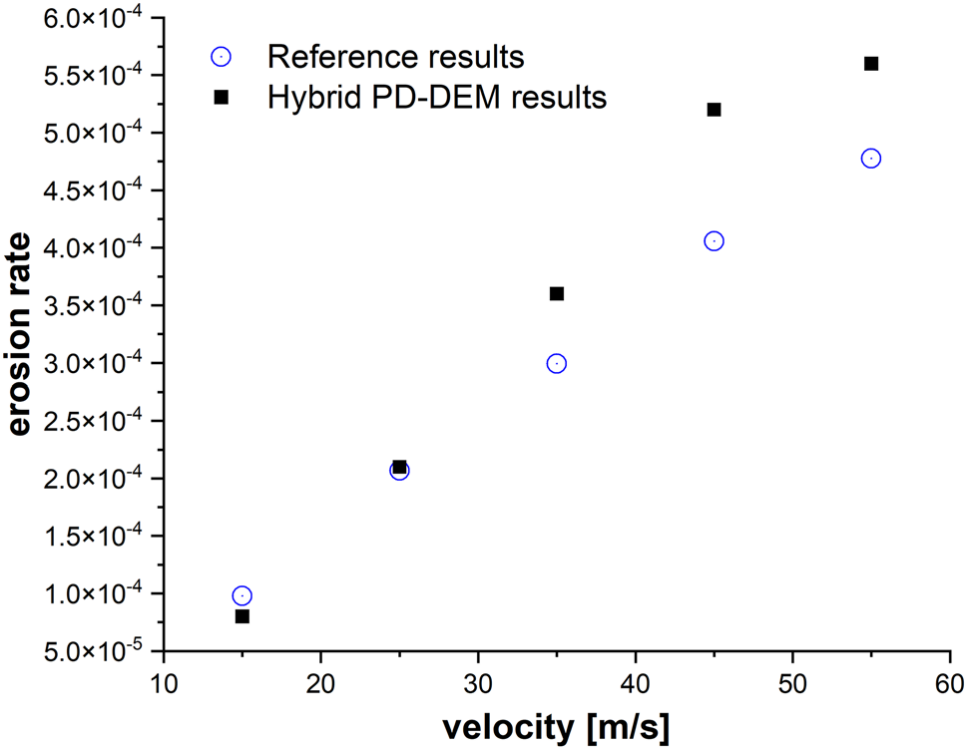
Correlation between erosion rate and impact velocity

In this validation study, we assess the erosion rate of the target material, comparing it against reference FEM results [59]. A spherical particle of radius $R = 0.2$ mm, described by DEM, is projected at an angle of 45° to the top surface of a cuboid-shaped target. The target is defined geometrically with dimensions: length $l = 5$ mm, width $w = 5$ mm and thickness $h = 4$ mm. The target cuboid is discretized using PD particles, each possessing a diameter $D = 0.1$ mm, as depicted in Fig. 5. Interactions between material points of the target cuboid are defined by bond-based PD with horizon length $\delta = 0.3$ mm and stretch constant $s_{0} = 0.1$ mm. Material parameters for both the sphere and the target are detailed in Table 2. The results obtained with the present hybrid PD–DEM model by considering the material points with damage index > 0.8 are graphically presented in Fig. 6, and a comparison with data from the literature [59] matches up quite well.

**Table 2:** Material properties

Object	Young’s modulus *E* (Pa)	Poisson’s ratio *v*	Density (kg/m^3^)
Sphere	70e9	0.3	2680
Cuboid	50e9	0.3	2600

## Wind turbine blade

From an engineering perspective, turbine blades are considered high-performance components due to their ability to withstand the operational loads and environmental conditions they are exposed to. When the blade speed is considered well above 80 m/s, LEE estimation becomes a critical design criterion. The design of wind turbine blades is an optimization between the need for structural strength and aerodynamic performance [60].

### Blade material

Modern wind turbine blades are made of composite laminates with glass or carbon fibers in polymeric resins [61]. These materials provide a high stiffness-to-weight ratio, fracture toughness and fatigue strength. However, they are vulnerable to transverse impact stresses [62]. To protect against environmental exposure, coating materials are applied to the outer surface of the blades. Blade manufacturers use two types of coating: epoxy/polyester-based gelcoat applied during manufacturing [63, 64] or flexible polyurethane coating/leading edge protection tape applied afterward [64, 65]. Delamination or debonding between the coating and substrate can accelerate leading edge erosion.

### Leading edge geometry

**Fig. 7:**
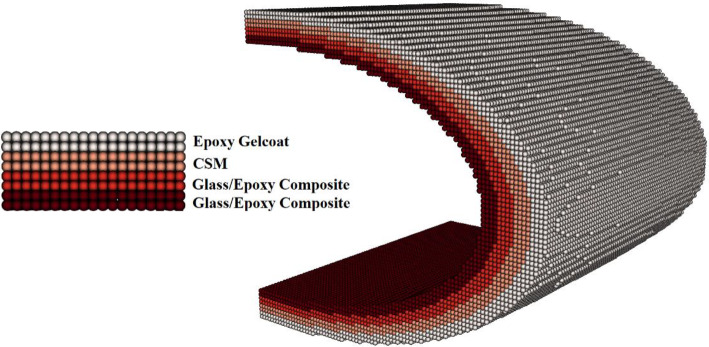
Section of leading edge profile geometry of wind turbine blade and material layout

The section of a leading edge profile geometry and the material layup configuration of a utility-scale blade tip is shown in Fig. 7. The layup configuration of the laminate is comprised of an epoxy gelcoat layer, an epoxy/glass chopped strand mat (CSM) layer and two layers of glass/epoxy composite, where the thickness of each layer is 2 mm. The dimensions of the leading edge, which have been chosen to shape as a parabolic cylinder, were thoughtfully selected to replicate a scaled-down cross-sectional profile commonly found in wind turbine blades. The material properties of the glass/epoxy composite and the two top protective layers are given in Table 3. These material properties are only considered to be a rough approximation of the typical material characteristics because they were obtained from different sources [66, 67]. The section of the blade is discretized considering simple cubic lattice with lattice constant equal to the diameter of PD particles.

**Table 3:** Material properties of the layers of laminate

Material	Epoxy gelcoat	CSM	Glass/epoxy composite
Density $\rho$ (kg/m^3^)	1400	1100	2540
Young’s modulus *E* (GPa)	7	3.5	26
Poisson’s ratio $\nu$	0.33	0.33	0.25
Mass of single PD particle (kg)	9.163e−8	7.199e−8	1.662e−7

## Results and discussion

### Sand particle impact and leading edge erosion

In this case, damage propagation is modeled in a section of the leading edge profile shown in Fig. 7 due to the normal impact of a solid sand particle. The target is shaped as a parabolic cylinder which represents the section of a leading edge profile of a wind turbine blade with *x* = − 20 mm…20 mm, *y* = − 20 mm…20 mm and *z* = 0…40 mm, where it is discretized with PD particles of radius 0.00025 m resulting in a total of 157,160 particles. The blade material layup configuration is also illustrated in Fig. 7, and the material properties of the glass/epoxy composite and the two top protective layers of epoxy gelcoat and CSM are given in Table 3. The spherical solid sand particle of diameter *D* = 2 mm described by DEM is projected normally at the center of the leading edge of the blade section with velocity **v** = − 70 m/s. The adaptive time settings are considered with maximum time step size Δ*t* = 1.0 × 10^−8^ s and total time *t* = 400 µs. The material properties of the impactor (solid sand particle) are listed in Table 4.

**Table 4:** Material properties of the sand particle

Material	Sand
Density $\rho$ (kg/m^3^)	2650
Young’s modulus *E* (GPa)	90
Poisson’s ratio $\nu$	0.2

**Fig. 8:**
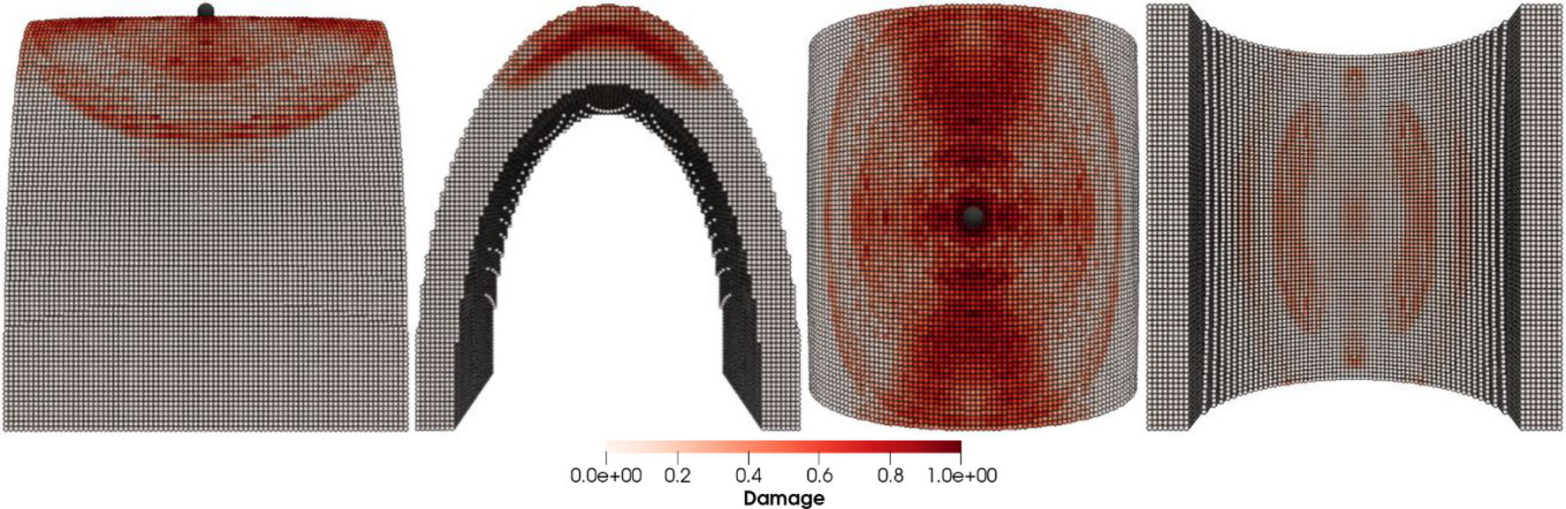
Contours of damage caused to the leading edge profile of wind turbine blade due to the impact of a sand particle of diameter 2 mm at an impact velocity of − 70 m/s

**Fig. 9:**
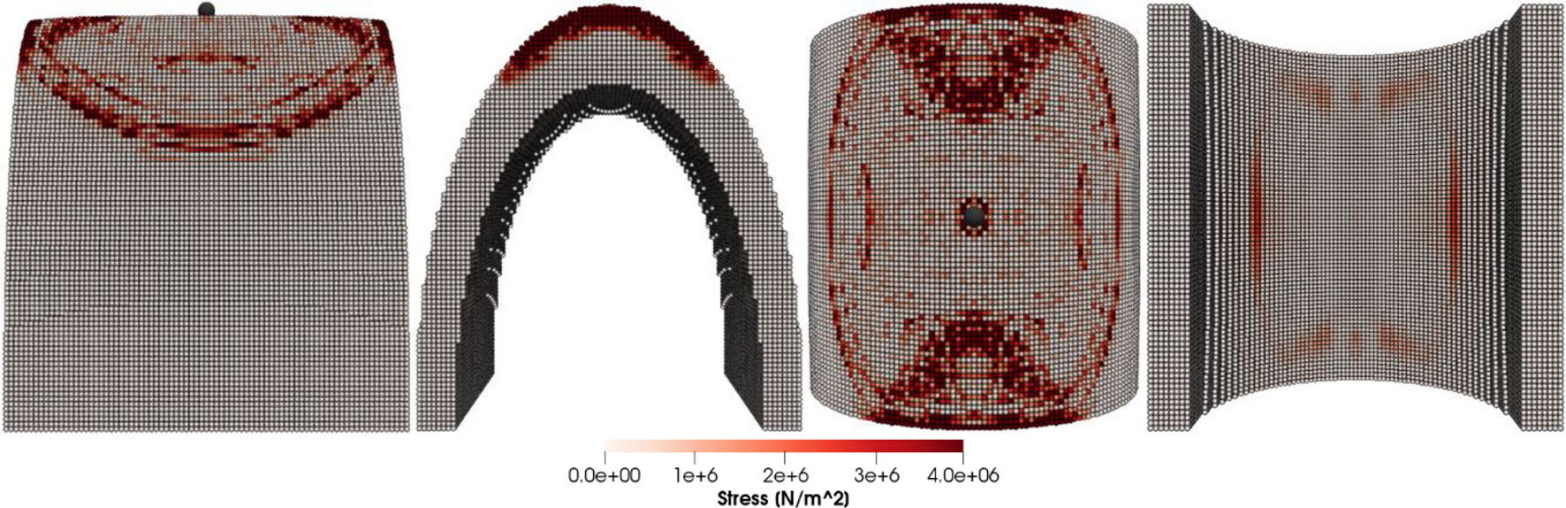
Contours of stress exerted on the leading edge profile of wind turbine blade due to the impact of a sand particle of diameter 2 mm at an impact velocity of − 70 m/s

**Fig. 10:**
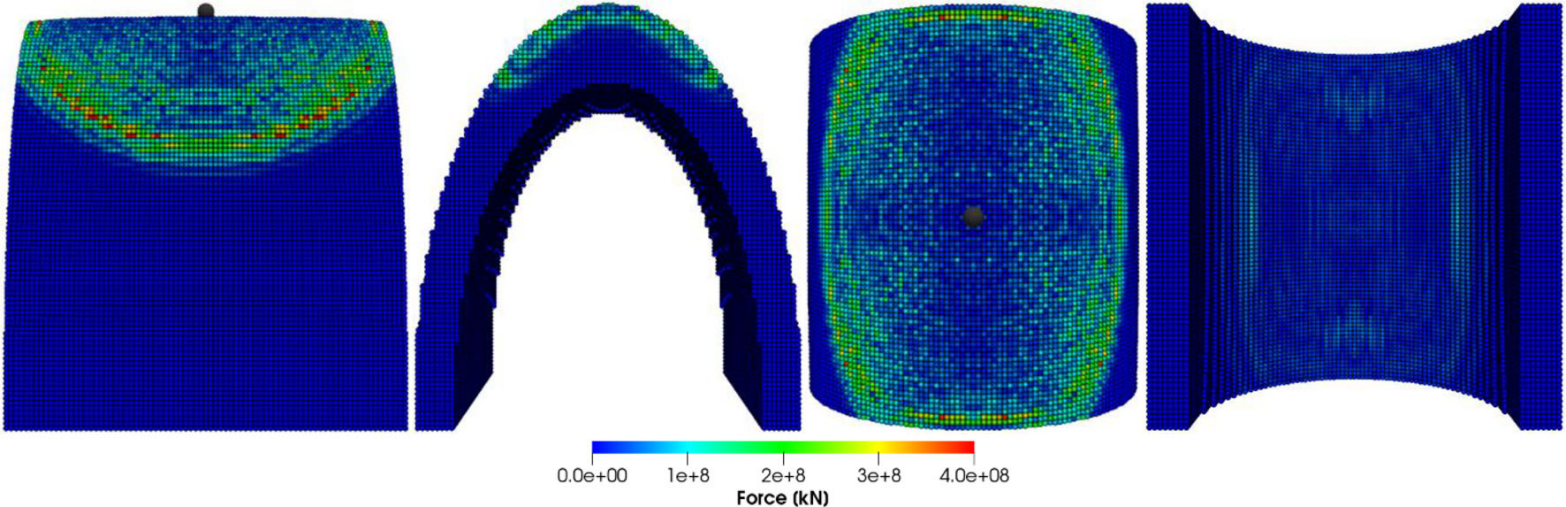
Contours of force exerted on the leading edge profile of wind turbine blade due to the impact of a sand particle of diameter 2 mm at an impact velocity of − 70 m/s

**Fig. 11:**
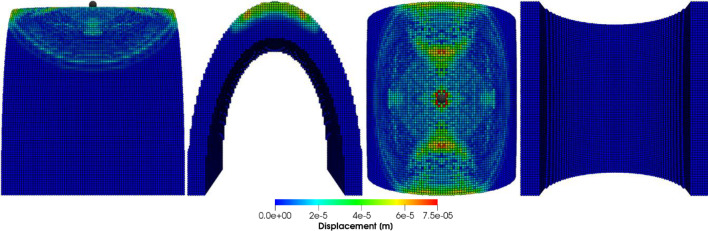
Contours of displacement of the material points of the leading edge profile of wind turbine blade due to the impact of a sand particle of diameter 2 mm at an impact velocity of − 70 m/s

**Fig. 12:**
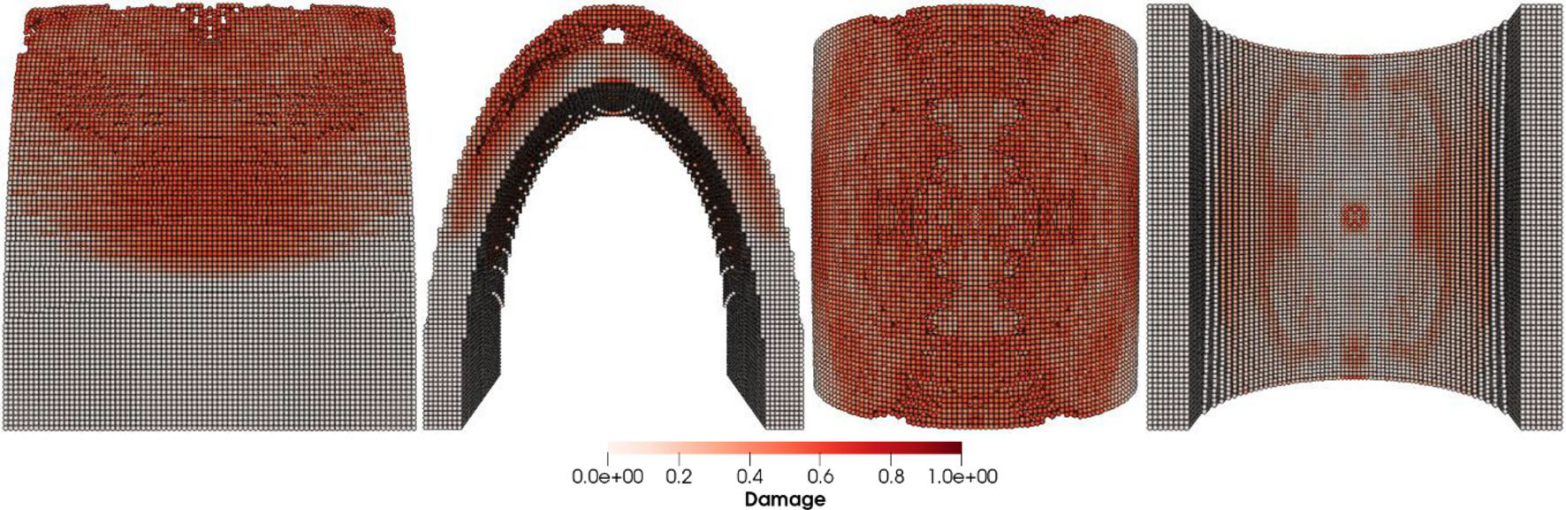
Delamination and damage patterns caused to the leading edge profile of the wind turbine blade after removing the material points with a damage index > 0.8

**Fig. 13:**
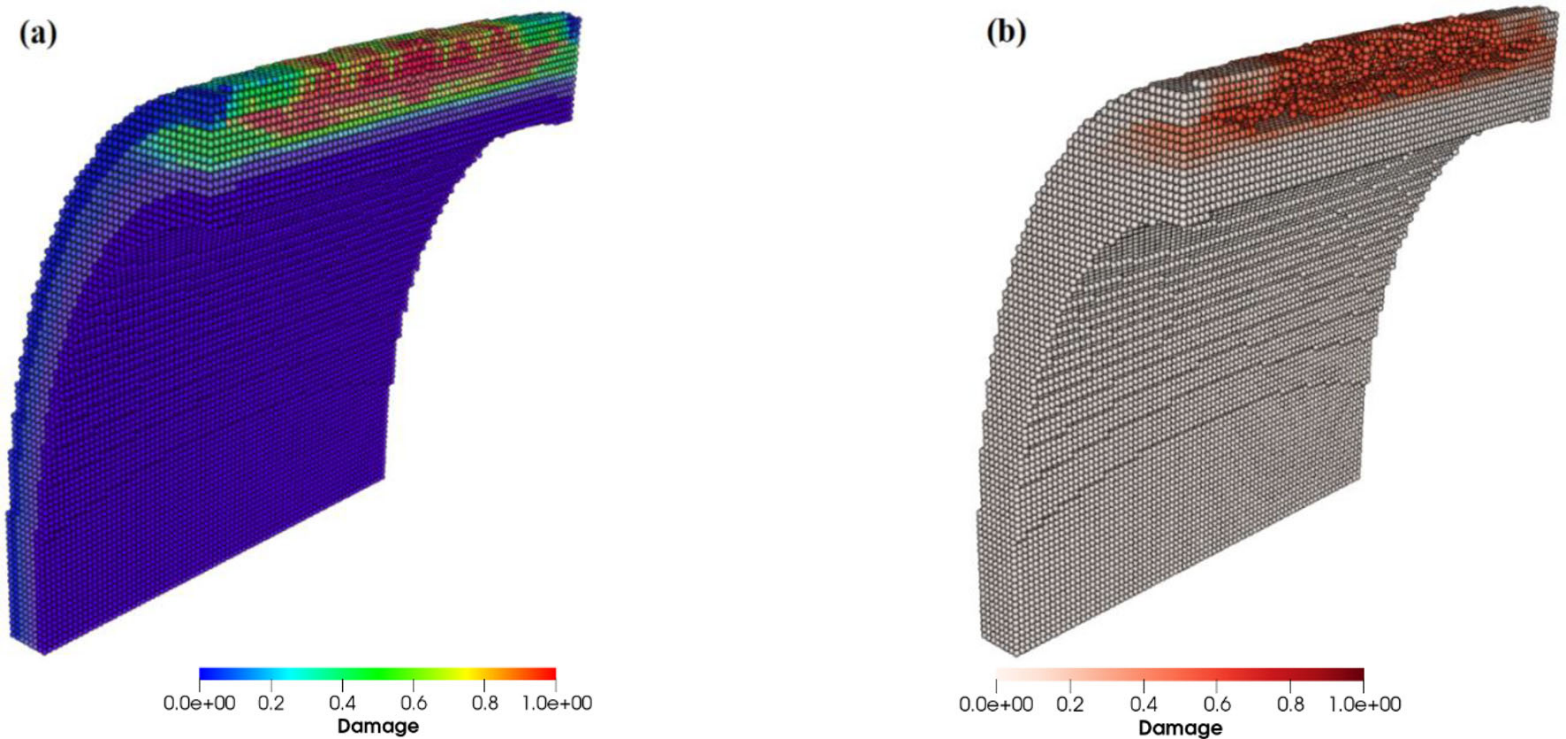
Cross-sectional view of the damaged blade showing **a** delamination and damage caused across laminate interfaces, **b** delamination and damage caused to the leading edge profile of wind turbine blade after removing the material points with damage index > 0.8

**Fig. 14:**
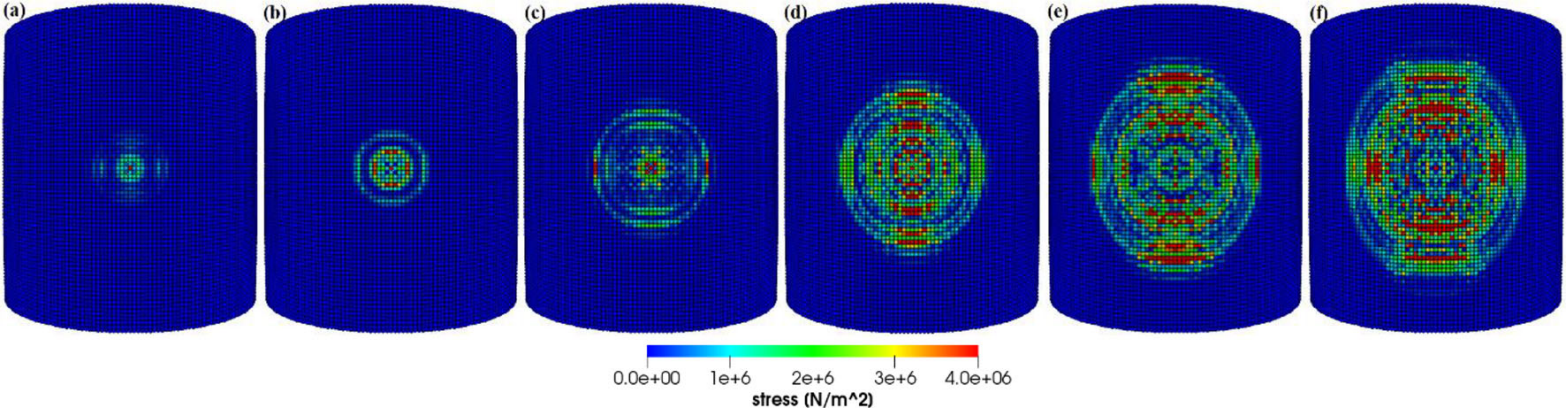
Concentric high-stress rings transmit energy to other regions of the blade at different time intervals from **a** to **f**, respectively

Figures 8, 9, 10 and 11 show the images of the damaged blade taken at various angles, illustrating the damage contours, stress contours, impact force contours and displacement contours of the target material points of the blade, respectively. The contour plots in Fig. 9 evidently show that stress propagates in the laminated material of the blade as concentric high-stress rings. There is a large intermediate stress between these stress bands, demonstrating that the formation of stresses in the coating is caused by the basic compressional impact behavior in the direction of impact. These stress distribution patterns indicate that sand particle impact force is a contributing factor in the development of blade damage. Regarding the potential types of damage, the distribution of stress inside the laminate might cause a variety of material failures, including interlayer delamination or general material failure. Figures 12, 13 and 14a-f illustrate that the impact of the sand particles results in delamination across laminate interfaces and potentially damages the protective layers and provides additional evidence that instead of only the top layers, the blade section experiences impact damage throughout its thickness, showing prominent damage outlines in the inner layers. When addressing sand particle impact damage, the affected areas of the blade may not be limited to the upper layers only, because of the through-thickness force and stress absorption behavior of composite laminates.

### Impact velocity effect

**Fig. 15:**
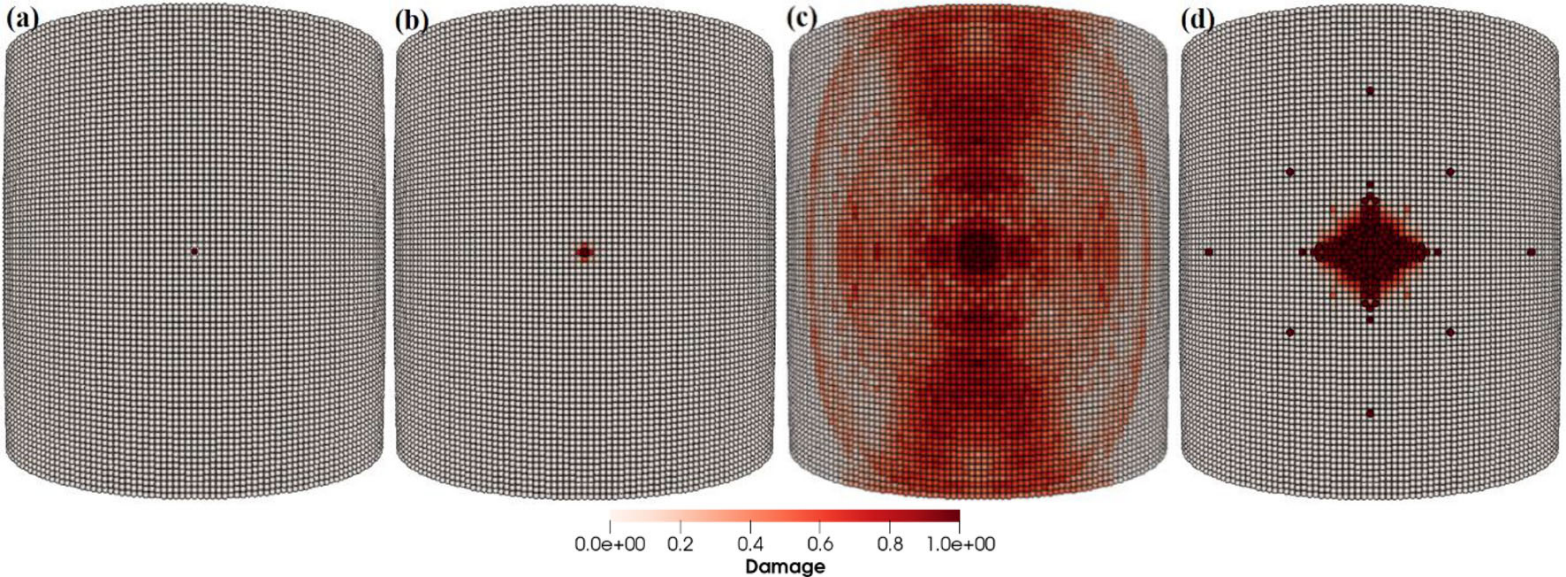
Contours of damage caused to the leading edge profile of wind turbine blade due to the impact of a sand particle of diameter 2 mm at an impact velocity of 30, 50, 70 and 100 m/s from **a** to **d**, respectively

**Fig. 16:**
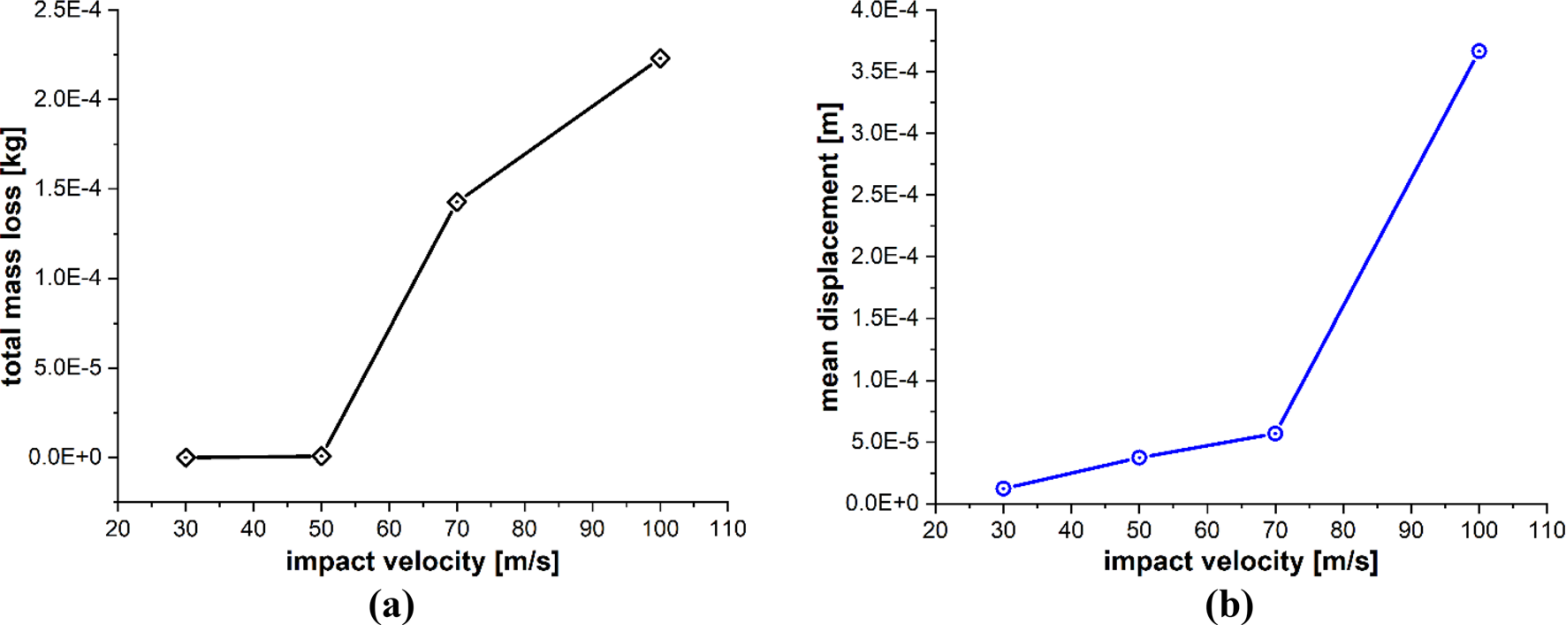
Positive correlation between the impact velocity and **a** total mass loss, **b** mean displacement of the target material points

**Fig. 17:**
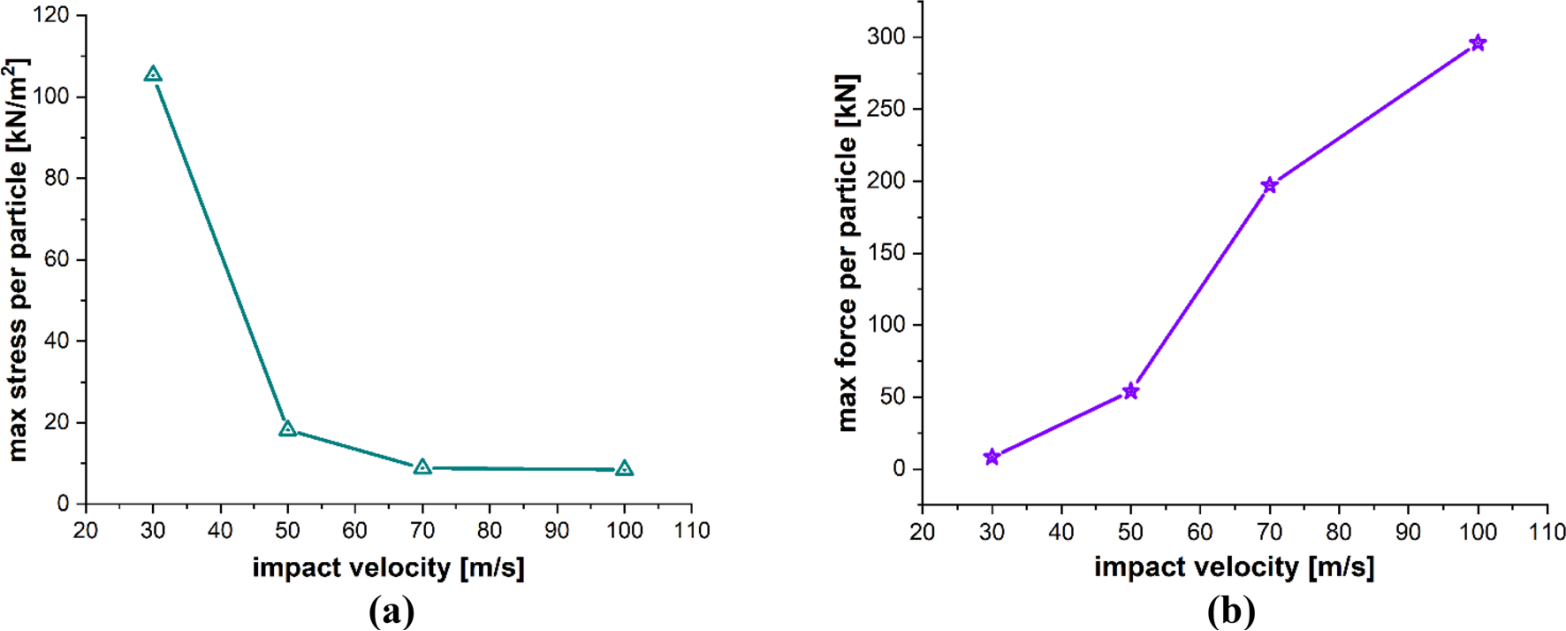
Negative and positive correlation, respectively, between particle impact velocity and **a** max stress per particle, **b** max force per particle on the target material points

The particle impact velocity is usually considered one of the most significant factors related to particle impact erosion. In this case, we investigate the effects of sand particle impact velocity on the LEE of wind turbine blades. The dimensions of the blade geometry and the laminates’ layup configuration, thickness and material characteristics are the same as those employed in the previous case. The spherical solid sand particle of diameter *D* = 2 mm described by DEM is projected normally at the center of the leading edge of the blade section with velocity **v** = − 30, − 50, − 100 m/s. The material properties of the sand particle are listed in Table 4. The maximum time step size is Δ*t* = 1.0 × 10^−8^ s, and the total simulation time is *t* = 1000 µs. Figure 15a–d depicts the patterns of damage contours at four different impact velocities, i.e., **v** = − 30, − 50, − 70 and − 100 m/s, respectively. The comparison of damage patterns in Fig. 15 reveals that the contours differ significantly as the impact velocity changes. At an impact velocity of **v** = − 30 and − 50 m/s, the sand particle causes small damage only at the point of immediate contact due to the relatively low energy of the incident particle and resulting in less impact force on the blade. However, the results at **v** = − 70 and − 100 m/s show significantly damaged regions further away from the point of contact of the sand particle. The impact force of the sand particle causes compressional effects on the blade surface in the direction of the impact. The impact force spreads to other regions of the blade through the laminated material by generating concentric high-stress rings as shown in Fig. 14, that transmit energy which breaks the bonds between material points and leads to the development of material damage in the blade. Figure 15 clearly shows that the damage to the blade is more widespread at an impact velocity of **v** = − 70 m/s than at **v** = − 70 m/s. It is therefore obvious that the damage is more confined at high impact velocities due to material failure in reaction to high impact force. The area that failed took the brunt of the impact energy and stops the compressional effects of the impact force, which halts the energy from spreading to other areas of the blade. Figure 16a, b and Fig. 17a, b provide detailed quantitative findings of the blade material response to the impact of the sand particle at different impact velocities. The plots in Fig. 16a and b show how changes in the impact velocity of the sand particle affect the amount of mass lost by the material and the degree to which the target material points are displaced, respectively. It is observed that as the impact velocity increases, so does the quantity of mass removed and the mean displacement of the target material points. This indicates a direct correlation between erosion rate and particle impact velocity. Figure 17a and b shows that the maximum impact force per particle increases as the impact velocity increases while the maximum stress per particle drops with an increase in velocity. This is because the material failure minimizes the compressional effects and vibrations brought on by the impact force. When particles lose their bonds, the stress on them is released, and they no longer contribute to the transmission of forces.

### Particle size effect

**Fig. 18:**
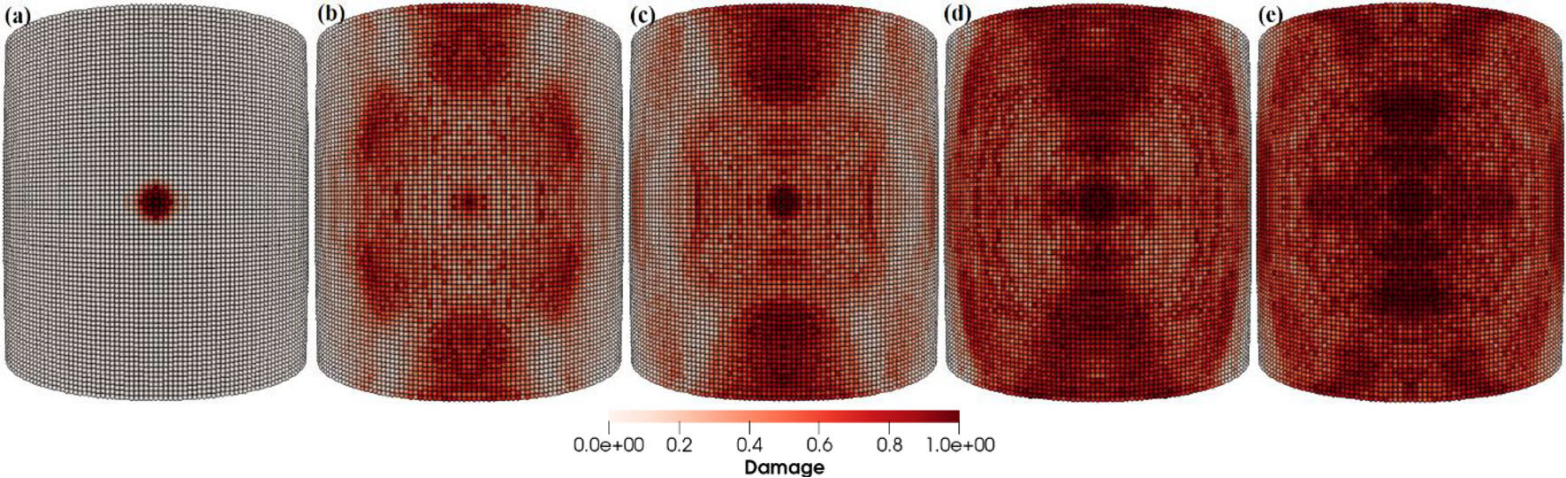
Contours of damage caused to the leading edge profile of wind turbine blade due to the impact of a sand particle of diameter 0.1, 0.5, 1, 2 and 5 mm at an impact velocity of 70 m/s from **a** to **e**, respectively

**Fig. 19:**
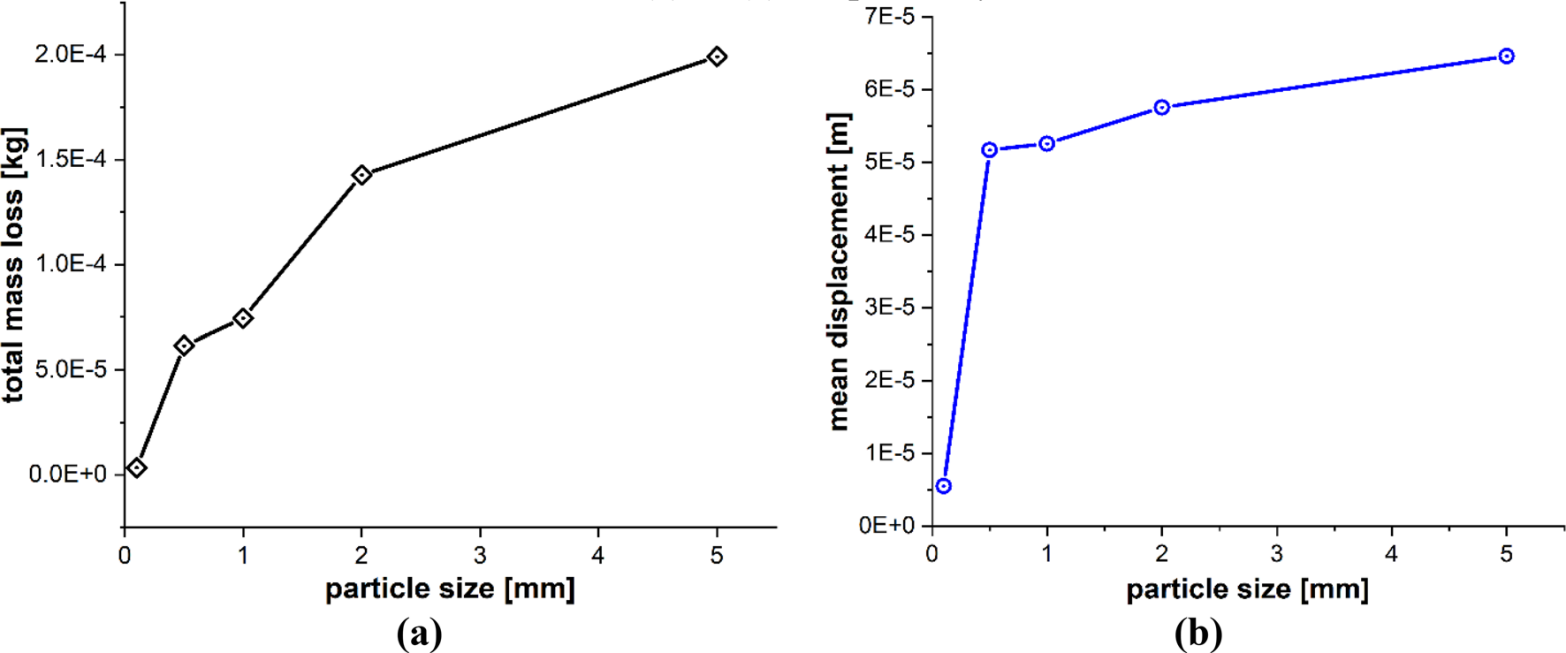
Positive correlation between the particle size and **a** total mass loss, **b** mean displacement of the target material points

**Fig. 20:**
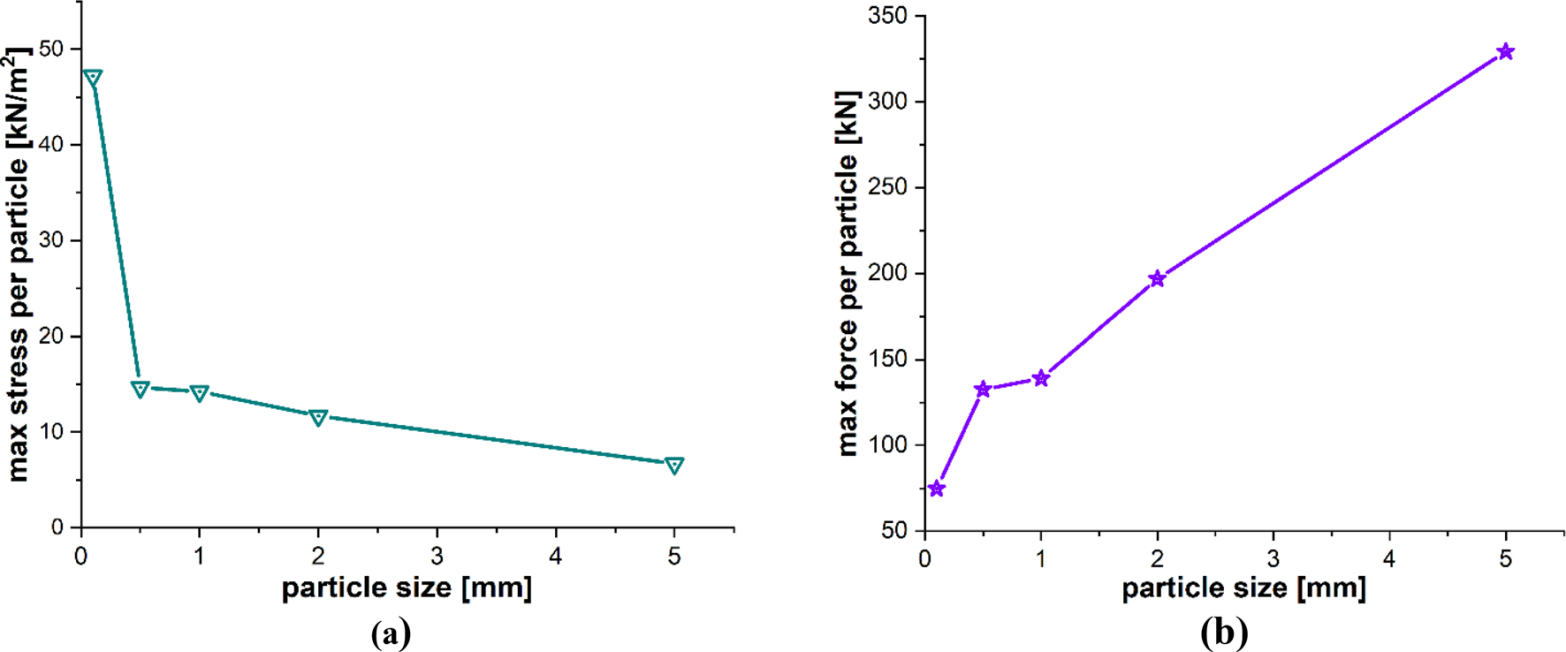
Negative and positive correlation, respectively, between particle size and **a** max stress per particle, **b** max force per particle on the target material points

The particle size is also a very influential parameter in the particle impact erosion process. In this case, simulations are carried out to understand the influence of particle size on the process of particle impact erosion of the leading edge of a wind turbine blade. The dimensions of the blade geometry and the laminates’ layup configuration, thickness and material characteristics are the same as those employed in the previous cases. The spherical solid sand particles of diameters *D* = 0.1, 0.5, 1, 2 and 5 mm described by DEM are projected normally at the center of the leading edge of the blade section with velocity **v** = − 70 m/s. The material properties of the sand particle are listed in Table 4. The maximum time step size Δ*t* = 1.0 × 10^−8^ s, and total time *t* = 400 µs. Figure 18a–e depicts the patterns of damage contours on the blade surface due to the impact of particles of five different sizes, i.e., *D* = 0.1, 0.5, 1, 2 and 5 mm at an impact velocity of **v** = − 70 m/s, respectively. The comparison of damage contours in Fig. 18 shows that when the particle size varies, the patterns of damage contours and damaged area differ significantly. When the impacting particle is *D* = 0.1 mm in diameter, the damage is more localized, while larger sizes result in more globalized damage patterns. The compressional effects of the particle impact force increase with increasing particle size, which spreads energy throughout the target and develops more material damage to the blade. Figures 19 and 20 provide detailed quantitative findings of the blade material response to the impact of sand particles of different sizes. Figure 19a and b demonstrates the impact of variations in sand particle size on the amount of mass lost by the material and the degree to which the target material points are displaced, respectively. This observation suggests a positive correlation between impact velocity and these two variables. As the impact velocity increases, both the mass removed and the mean displacement of the target material points show an upward trend. Figure 20a and b indicates that as the size of the incident particle increases, there is a corresponding increase in the maximum impact force per particle, while the maximum stress per particle decreases, respectively, because the material failure mitigates the compressional effects and vibrations induced by the impact force. When the incident particle size is *D* = 0.1 mm, it is considerably smaller than the target’s PD particles, which may account for the disparate impact force and stress values. Kinetic energy and velocity have a squared connection, i.e., $\text{K.E} = {1 \mathord{\left/ {\vphantom {1 2}} \right. \kern-0pt} 2}m{\mathbf{v}}^{2}$, whereas particle size $r$ and kinetic energy have a cubic relationship, i.e., $\text{K.E}. = {2 \mathord{\left/ {\vphantom {2 3}} \right. \kern-0pt} 3}\pi \rho r^{3} {\mathbf{v}}^{2}$. A little change in the particle size has a significant impact on the erosion rate. Therefore, the size of a particle is an important particle characteristic that influences erosion magnitude because bigger particles have more kinetic energy even when they strike the target with the same velocity as the smaller particles.

### Impact angle effect

Another important aspect that affects the intensity of particle impact erosion is the angle at which the particles strike the target. In this case, simulations are carried out to understand the influence of particle impact angle on the process of particle impact erosion of the leading edge of a wind turbine blade. The dimensions of the blade geometry and the laminates’ layup configuration, thickness and material characteristics are the same as those employed in the previous cases. The spherical solid sand particles of diameters $D = 2$ mm described by DEM are projected at the center of the leading edge of the blade section with velocity ${\mathbf{v}} = - 70$ m/s at six different angles to the blade surface, i.e., *θ* = 15°, 30°, 45°, 60°, 75° and 90°. The material properties of the sand particle are listed in Table 4. The maximum time step size Δ*t* = 1.0 × 10^−8^ s, and total time *t* = 1000 µs. Depending on the particle’s line of impact, we examine two different scenarios. In the first situation, the particle’s line of impact is considered to be perpendicular to the blade length, whereas in the second case, the line of impact is considered to be along the blade length.

#### Line of impact is perpendicular to blade length

**Fig. 21:**
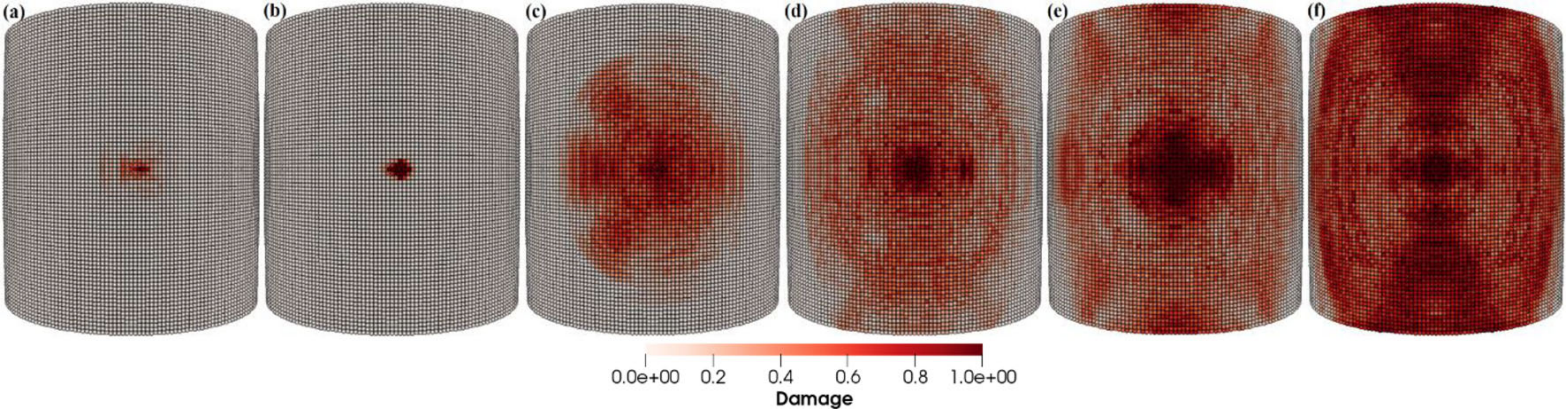
Contours of damage caused to the leading edge profile of wind turbine blade due to the impact of a sand particle of diameter 2 mm with an impact velocity of 70 m/s and at impact angles of 15°, 30°, 45°, 60°, 75° and 90° from **a** to **f**, respectively

In this case, the sand particle direction of impact creates angles perpendicular to the blade length. Figure 21a–f depicts the patterns of damage contours on the blade surface due to the impact of sand particles having impact velocity ${\mathbf{v}} = - 70$ m/s making six different impact angles to the blade surface, i.e., *θ* = 15°, 30°, 45°, 60°, 75° and 90°, respectively. The comparison of damage contours in Fig. 21 shows that the intensity of impact erosion varies with particle impact angle. It has been found that the blade experiences maximum erosion at the impact angles close to normal where cracking is the primary cause of erosion. Smaller impact angles cause more localized damage, whereas larger impact angles cause more widespread damage to the blade’s leading edge. The compressional effects of the particle impact force grow with increasing impact angle, which transfers energy throughout the target and causes more material damage to the blade. Additionally, impacts with angles below the normal create damage contours that are symmetrical across the line of impact, i.e., about the horizontal axis of the blade section.

#### Line of impact is along the blade length

**Fig. 22:**
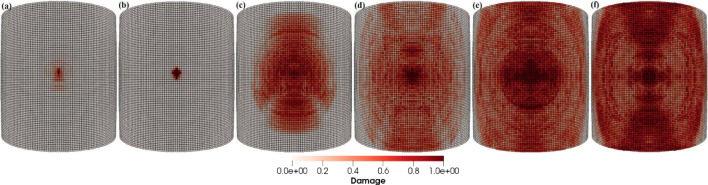
Contours of damage caused to the leading edge profile of wind turbine blade due to the impact of a sand particle of diameter 2 mm with an impact velocity of 70 m/s and at impact angles of 15°, 30°, 45°, 60°, 75° and 90° from **a** to **f**, respectively

In this case, the sand particle direction of impact creates angles with the blade axis that is parallel to the blade length. Figure 22a–f depicts the patterns of damage contours on the blade surface due to the impact of sand particles having impact velocity **v** = − 70 m/s making six different impact angles to the blade surface, i.e., *θ* = 15°, 30°, 45°, 60°, 75° and 90°, respectively. The comparison of the damage contours in Fig. 22 demonstrates how the particle impact angle affects the amount of impact erosion and it has been discovered that the blade erodes more rapidly at impact angles that are near to normal. Larger impact angles damage the leading edge of the blade more globally, whereas smaller impact angles cause more local damage. As the impact angle increases, the compressional effects of the particle impact force increase, transferring energy throughout the target and causing more material damage to the blade. Furthermore, particle impacts with angles less than normal produce damage contours that are symmetrical across the line of impact, i.e., about the blade axis which is parallel to the blade length.

**Fig. 23:**
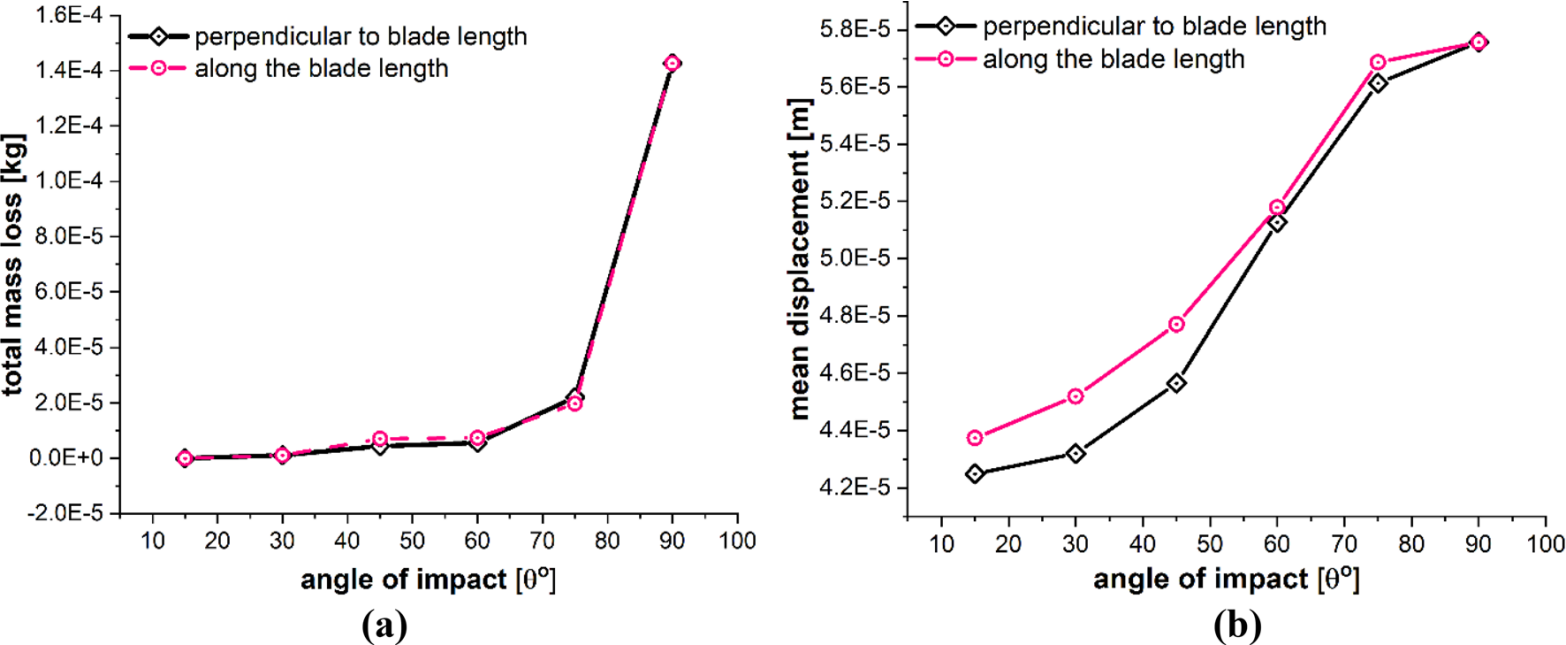
Effects of impact angle on **a** total mass loss, **b** mean displacement of the target material points

**Fig. 24:**
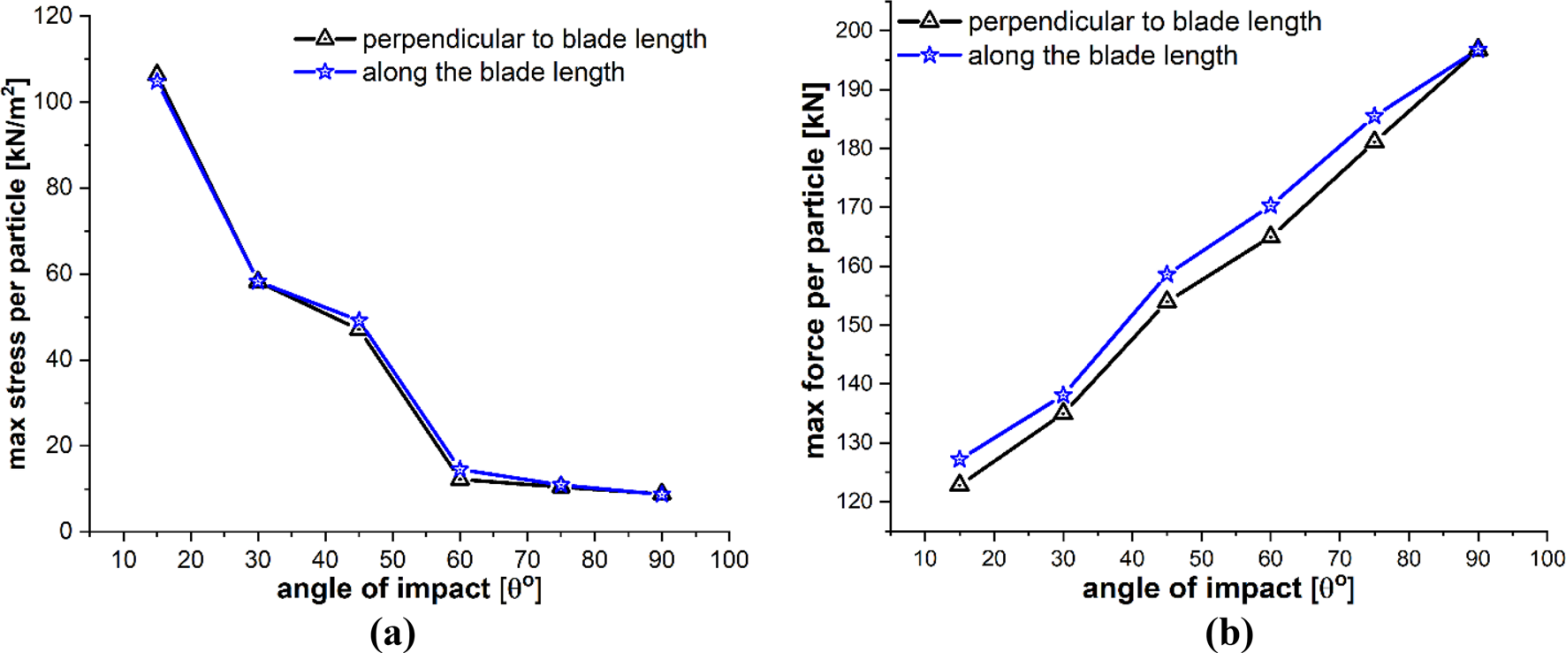
Effects of impact angle on **a** max stress per particle, **b** max force per particle on the target material points

The detailed quantitative results of the blade material response to the impact of sand particles at different impact angles are presented in Figs. 23 and 24. Figure 23a and b shows the effects of variations in the impacting angle of sand particles on the amount of mass lost by the target material and the degree to which target material points are displaced, respectively. The results indicate that both the quantity of mass removed and the mean displacement of the target material points increase as the impact angle increases. These values reach their maximum when the impact angle approaches to normal. Figure 24a and b shows that the particle impact angle increases, and so does the maximum force per particle, but the maximum stress per particle decreases as the impact angle approaches to normal. This occurs because material failure reduces the compressional effects and vibrations caused by the impact force. As particles lose their bonds, the stress is released, rendering them incapable of further transmitting forces. The plots in Figs. 23 and 24 also provide a comparative analysis of the results for the two different lines of action of the impacting particle, i.e., perpendicular to the blade length and along the blade length. Overall, the results of both cases are quite similar, with only a slight difference observed in the displacement of material points when the impact angle is small.

#### Impacts on the side of leading edge

**Fig. 25:**
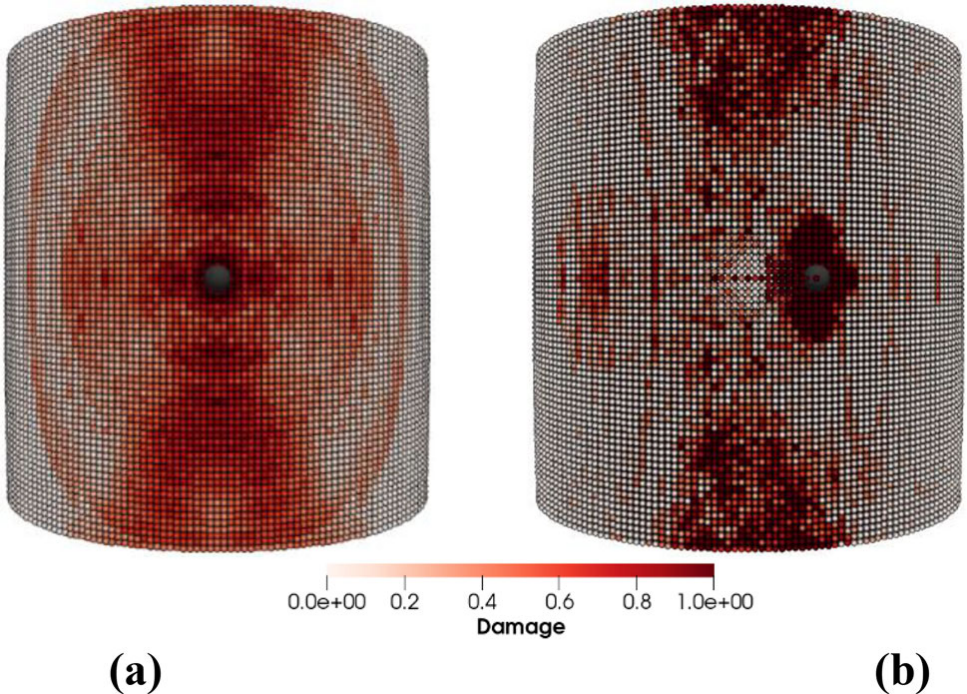
Contours of damage caused to the wind turbine blade due to the impact of a sand particle of diameter 2 mm with an impact velocity of 70 m/s **a** impacting on the leading edge and **b** impacting slightly to the side of the leading edge

The objective of this case study is to distinguish our analysis from the examination of impacts on flat surfaces. We achieve this by simulating impacts slightly off the leading edge on the curved surface of the blade. The direction of impact of sand particle of diameters $D = 2$ mm creates angles perpendicular to the blade length, we consider six different angles to the blade surface, i.e., *θ* = 15°, 30°, 45°, 60°, 75° and 90°. In Fig. 25a and b, we illustrate the patterns of damage contours resulting from impacting sand particles on both the leading edge and slightly to the side of it, with an impact velocity of **v** = − 70 m/s. Comparing the damage contours in Fig. 25, it becomes evident that the intensity of impact erosion varies depending on the point of impact on the blade’s surface.

**Fig. 26:**
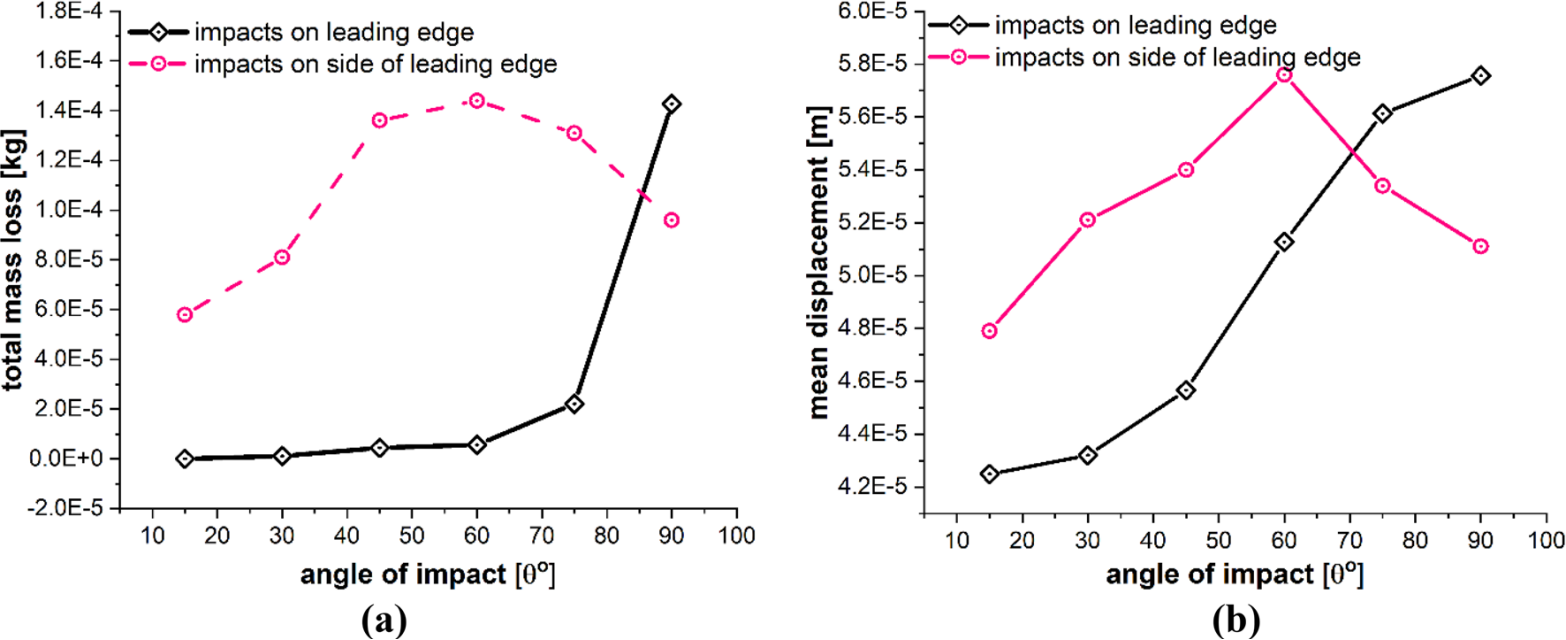
Effects of impact position and impact angle on **a** total mass loss, **b** mean displacement of the target material points

**Fig. 27:**
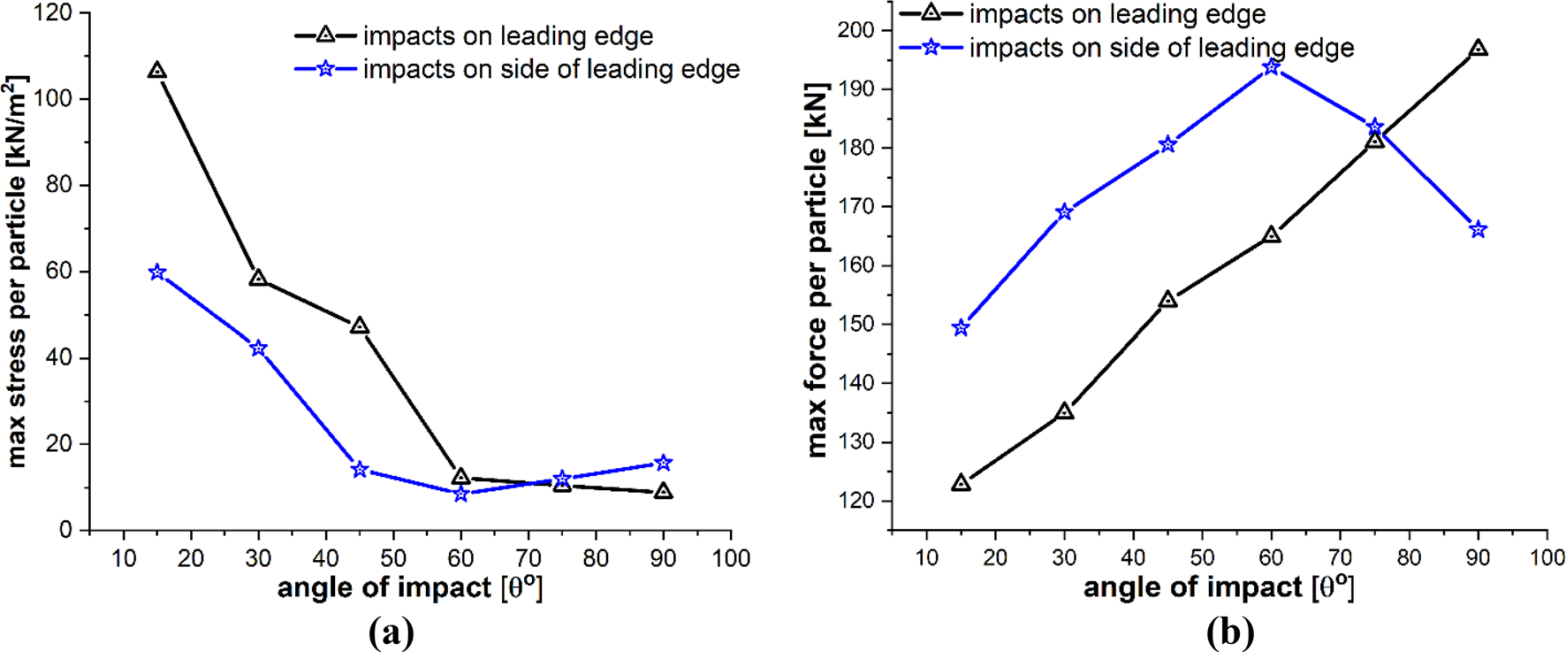
Effects of impact position and impact angle on **a** max stress per particle, **b** max force per particle on the target material points

Comprehensive quantitative results concerning the blade material’s response to sand particle impact at two distinct positions (directly on the leading edge and slightly off-center from the leading edge) and at various impact angles are displayed in Figs. 26 and 27. Figure 26a and b illustrates the effects of changing sand particle impact angles and impact position on the mass loss incurred by the target material and the displacement of target material points, respectively. Figure 27a provides a comparison of the maximum force per particle, while Fig. 27b illustrates the comparison of maximum stress per particle. These data represent the impact outcomes for both impact positions at various impact angles. The comparative analysis of the results depicted in Figs. 26 and 27, representing two distinct impact positions and varying impact angles, clearly demonstrates divergent material behaviors. The results relates significant influence of the specific point of impact on the particle impact erosion of WTB. It is important to clarify that when employing an impactor size (e.g., diameter $D \le 1$ mm) significantly smaller than the geometry of the leading edge, we have observed that the impact position has minimal influence on both the magnitude of the impact force and the amount of mass removed.

### Multiple particle impacts

**Fig. 28:**
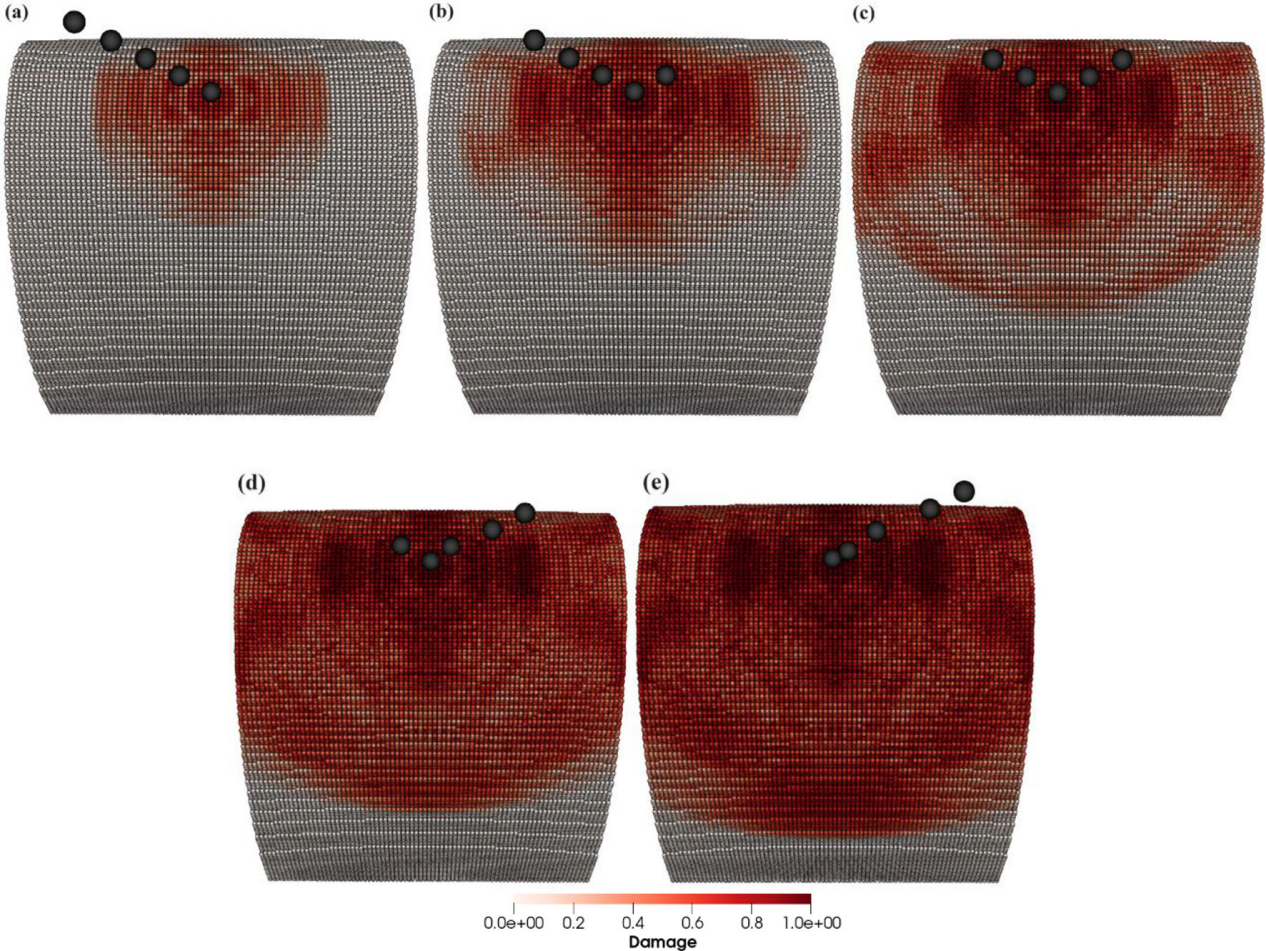
Contours of damage caused to the wind turbine blade due to the impacts of 5 sand particles of diameter 2 mm with an impact velocity of 60 m/s from **a** to **e**, respectively

The aim of this case is to simulate and study multiple particles’ impact on the leading edge of the wind turbine blade. It is achieved by simulating the impacts of five particles at the same point on the leading edge. The incident stream of sand particles of diameter $D = 2$ mm creates an angle of $\theta = 45^{ \circ }$ relative to the blade length and has impact velocity of $v = 60$ m/s. Figure 28a–e illustrates the damage contour patterns on the blade surface resulting from the repeated impacts of sand particles. The images depicting damaged contours in Fig. 28 clearly shows a substantial increase in both damage contours and the affected area as the number of impacts increase.

**Fig. 29:**
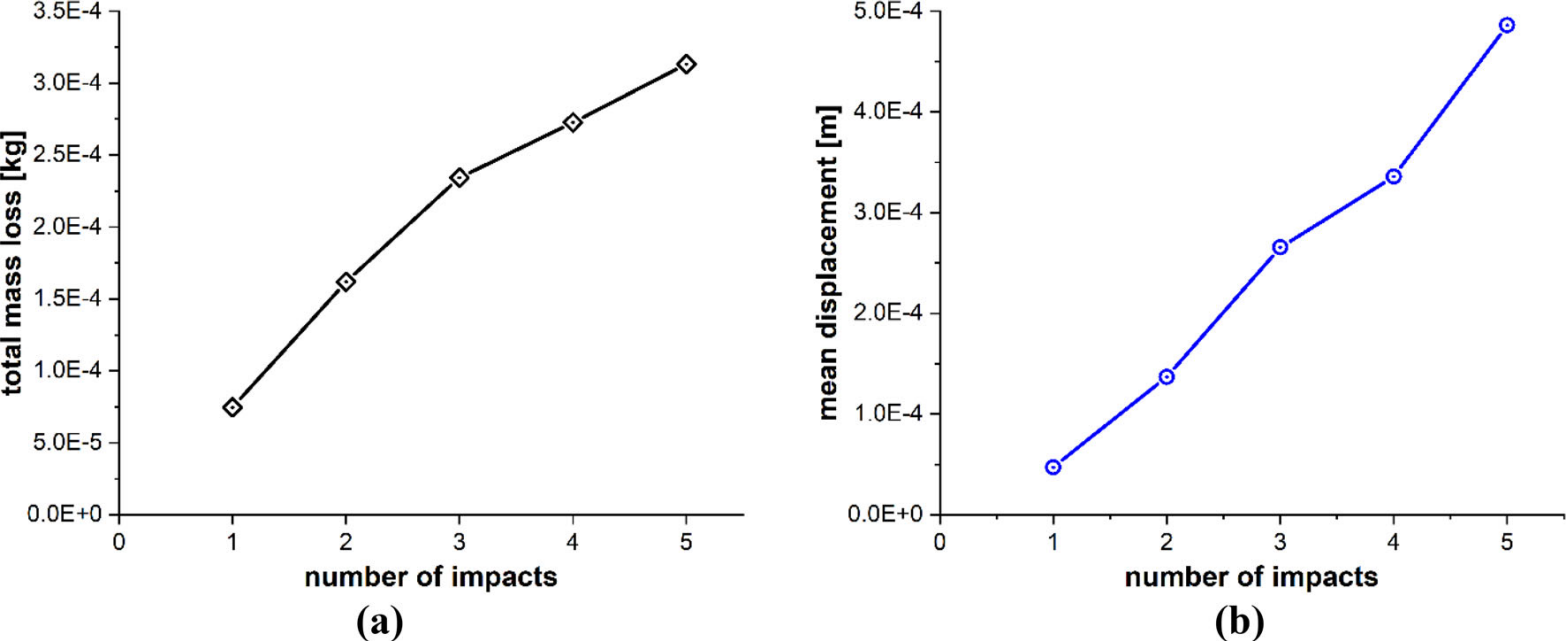
Positive correlation between the number of impacts and **a** total mass loss, **b** mean displacement of the target material points

Figure 29a and b presents a detailed quantitative insights into how the blade material responds to the varying impact numbers of sand particles. Figure 29a illustrates the effects of the number of sand particle impacts on the mass loss of the target material, while Fig. 29b focuses on the displacement of target material points. The data suggests a positive correlation between the number of impacts and these two variables. With an increasing number of impacts, both the mass removed and the mean displacement of the target material points exhibit an upward trend. Consequently, we conclude that the leading edge erosion of the wind turbine blade at a specific point is contingent on the number of impacting particles at that specific point.

## Conclusions

This paper employs a particle-based hybrid approach that combines the peridynamics theory with the DEM, in order to simulate particle impact events and model leading edge erosion of wind turbine blades caused by colliding sand particles. In this coupled framework, the force–displacement relations provided by Hertz and Mindlin in the normal and tangential directions, respectively, are used to model the particle interaction with the target material. The contact model also includes features such as intra-particle stiffness, damping effects and contact friction which are typically overlooked in repulsive force models used in PD simulations. The hybrid PD–DEM model underwent rigorous testing and validation in our earlier research [49], specifically focusing on contact parameters and resulting damage. The comprehensive validation involved extensive qualitative and quantitative comparisons with experimental and numerical data found within the existing literature. The erosion brought on by an impinging sand particle at the leading edge of a WTB is studied using the current approach, and the influence of erosive particle-related parameters such as particle size, impact velocity, impact angle, impact position and number of impacts at a point is systematically examined. The sand particle’s force of impact compresses the blade surface in the direction of the impact. The impact force propagates away from the point of particle contact through the laminated material by producing concentric high-stress rings that transport energy, breaking the bonds between material points and causing material damage in the form of interlayer delamination or general material failure. It is observed that the force of the impact increases as the impact velocity, particle size and number of impacts increase and the impact angle gets closer to normal. The amount of mass removed and the mean displacement of the target material points both increases with increasing impact force. Furthermore, it has been observed that the particle impact position on the WTBs geometry also has a substantial influence on the erosion mechanism. A substantial improvement in erosion prediction capability is presented in this study, which will advance WTB design and maintenance for the effective mitigation of LEE.

## Footnotes

1 Large-scale Atomic/Molecular Massively Parallel Simulator.
